# Differential susceptibility of *Onchocerca volvulus* microfilaria to ivermectin in two areas of contrasting history of mass drug administration in Cameroon: relevance of microscopy and molecular techniques for the monitoring of skin microfilarial repopulation within six months of direct observed treatment

**DOI:** 10.1186/s12879-020-05444-2

**Published:** 2020-10-02

**Authors:** Raphael Awah Abong, Glory N. Amambo, Patrick W. Chounna Ndongmo, Abdel Jelil Njouendou, Ritter Manuel, Amuam Andrew Beng, Mathias Eyong Esum, Kebede Deribe, Jerome Fru-Cho, Fanny F. Fombad, Theobald Mue Nji, Peter Ivo Enyong, Catherine B. Poole, Kenneth Pfarr, Achim Hoerauf, Clotilde K. S. Carlow, Samuel Wanji

**Affiliations:** 1 Parasites and Vector Research Unit (PAVRU), Department of Microbiology and Parasitology, University of Buea, P.O. Box 63, Buea, Cameroon; 2 Research Foundation in Tropical Diseases and Environment (REFOTDE), P.O. Box 474, Buea, Cameroon; 3 Department of Biomedical science, Faculty of Health Sciences, University of Buea, P.O. Box 63, Buea, Cameroon; 4 Institute for Medical Microbiology, Immunology and Parasitology, University Hospital Bonn, Bonn, Germany; 5 Global Health and Infection Department, Brighton and Sussex Medical School, Brighton, BN1 9PX UK; 6 School of Public Health, Addis Ababa University, Addis Ababa, Ethiopia; 7 Department of Sociology and Anthropology, University of Buea, Buea, Cameroon; 8 New England Biolabs, Ipswich, MA USA; 9 German Center for Infection Research (DZIF), partner site Bonn-Cologne, Bonn, Germany

**Keywords:** Susceptibility, Monitoring, *O. volvulus*, Microfilaridemia, Microscopy, Real-time PCR, LAMP, Microfilaricides

## Abstract

**Background:**

Ivermectin is an excellent microfilaricide against *Onchocerca volvulus.* However, in some regions, long term use of ivermectin has resulted in sub-optimal responses to the treatment. More data to properly document the phenomenon in various contexts of ivermectin mass drug administration (IVM-MDA) is needed. Also, there is a need to accurately monitor a possible repopulation of skin by microfilariae following treatment. Skin snip microscopy is known to have a low sensitivity in individuals with light infections, which can be the case following treatment. This study was designed with two complementary objectives: (i) to assess the susceptibility of *O. volvulus* microfilariae to ivermectin in two areas undergoing IVM-MDA for different lengths of time, and (ii) to document the repopulation of skin by the *O. volvulus* microfilariae following treatment, using 3 independent diagnostic techniques.

**Method:**

Identified microfilaridermic individuals were treated with ivermectin and re-examined after 1, 3, and 6 months using microscopy, actin real-time PCR (actin-qPCR) and O-150 LAMP assays. Susceptibility to ivermectin and trends in detecting reappearance of skin microfilariae were determined using three techniques. Microscopy was used as an imperfect gold standard to determine the performance of actin-qPCR and LAMP.

**Results:**

In Bafia with over 20 years of IVM-MDA, 11/51 (21.6%) direct observe treated microfilaridemic participants were still positive for skin microfilariae after 1 month. In Melong, with 10 years of IVM-MDA, 2/29 (6.9%) treated participants were still positive. The microfilarial density reduction per skin biopsy within one month following treatment was significantly lower in participants from Bafia.

In both study sites, the molecular techniques detected higher proportions of infected individuals than microscopy at all monitoring time points. LAMP demonstrated the highest levels of sensitivity and real-time PCR was found to have the highest specificity.

**Conclusion:**

Patterns in skin mirofilariae clearance and repopulation were established. *O*. *volvulus* worms from Bafia with higher number of annual MDA displayed a lower clearance and higher repopulation rate after treatment with ivermectin. Molecular assays displayed higher sensitivity in monitoring *O. volvulus* microfilaridemia within six months following treatment.

## Background

Onchocerciasis (river blindness) is a disease caused by infection with the filarial nematode *Onchocerca volvulus* and transmitted by female blackflies of the genus *Simulium*. It is one of the neglected tropical diseases with an estimated 21 million people infected, 99% of which are in Africa [1]. Infection with the parasite is known to be responsible for severe skin and ocular manifestations [2–5]. Ivermectin is the drug that is currently used for onchocerciasis control through mass administration in endemic areas. It is known that a single standard dose (150 μg/kg body weight) of ivermectin can result in 98–99% reduction in skin microfilariae (Mf) within one month of treatment [6].. Repopulation of skin by microfilariae occurs slowly, starting 3 months after treatment, and by 6 and 12 months after a single ivermectin dose, the Mf load is expected to have recovered to approximately 10 and 20% of pre-treatment levels, respectively [6]. A suboptimal response by parasites to ivermectin will lead to a slow rate of Mf reduction as well as a faster rate of Mf repopulation after treatment with a standard dose of ivermectin. Faster rates of skin repopulation have been reported in areas where parasites have experienced long term exposure to ivermectin (over 12 rounds of ivermectin mass drug administration (IVM-MDA) when compared to areas naïve to treatment [7, 8]. In contrast to previous studies where individuals from naïve endemic areas were compared to those from areas with long term exposure [7–9], participants in this study were recruited from two areas with contrasting histories of IVM-MDA to determine the effects of exposure to one or two decades of treatment on parasite susceptibility to drug. Ivermectin therapeutic coverage between 2010 and 2014 was at least 75% (75–80%) in both community directed treatment with ivermectin (CDTI) project areas in Centre 1 and Littoral 2 [10] and remained above 80% during the 2015, 2016 and 2017 treatment periods.

Microscopic detection and identification of Mf, based on morphological characteristics [11, 12] in skin snips remains the gold standard for the diagnosis of onchocerciasis in humans [13, 14]. However, previous findings have raised questions about the reliability of this method due to the presence of Mf of other species which can also reside in the skin. In one study, *Mansonella streptocerca* Mf in skin snips using PCR techniques, raised doubts about the specificity of microscope-based diagnosis of *O. volvulus* infection [15]. Similar concerns were reported recently following the detection of *Loa loa* Mf in skin snips in Cameroon [16]. Moreover, microscopy may not be able to detect infection in patients with very low microfilaridermia [11]. In addition, the insufficient sensitivity of skin snip microscopy has been demonstrated in a report from Uganda and Ethiopia where 84 of 853 samples that were declared negative by microscopy were positive using polymerase chain reaction (PCR) and Melt-Curve Analysis (PCR-MCA) [12]. The low sensitivity of microscopy can be more pronounced when people are treated with a microfilaricide which will further lower the Mf density [11, 12]. Conclusively, microscopy could be defined as an imperfect gold standard. Thus, the suboptimal sensitivity of classical microscopy inherently provides inaccurate information for modelers and therefore, existing predictions (ONCHOSIM and EPIONCHO) for the national programmes to evaluate the progress towards elimination of transmission were based mainly on microscopic examination and might be insufficient and too optimistic [17]. Therefore, there is an urgent need to apply accurate, simple and affordable diagnostic tools to detect low *O. volvulus* infection levels in humans during monitoring and evaluation of clinical trials as well as control and elimination programs.

Besides microscopy [11], immunological [18, 19] and nucleic acid-based methods, specifically PCR [20, 21] and LAMP [22, 23] have been used to diagnose *O. volvulus* in skin. Although there have been advances in the development of immunological methods involving detection of either antibody or antigen [18, 19], cross-reactivity with other filarial species has been reported [24]. The specificity for *O. volvulus* requires the use of a probe [25]. PCR assays have been developed for microfilariae detection in skin biopsies using different DNA targets including the *Onchocerca*-specific tandemly repeated DNA sequence family with a unit length of 150 base pairs (O-150) [12, 26, 27] and Ov Actin [28–30]. However, PCR-based methods can be time consuming, require highly purified DNA and require expensive equipment and material compared to microscopy. Nevertheless, this technology was shown to be more sensitive than microscopy [12, 21]. Alternatively, loop-mediated isothermal amplification (LAMP) is a simple molecular method which rapidly synthesizes large amounts of DNA within 60 min and which is less sensitive to DNA impurities and has higher amplification efficiency than PCR [22, 23, 31–34]. In addition, LAMP does not require any expensive equipment and thus it is easier to use in low resource-settings [35–38]. LAMP assays targeting O-150 and the mitochondrial *cox*1 gene have been established for the diagnosis of onchocerciasis [22, 23, 36].

The purpose of this study was to decipher two complementary objectives: (i) to assess the susceptibility of *O. volvulus* Mf to IVM in two regions that have undergone IVM-MDA for different time periods, and (ii) to document the repopulation of skin by the *O. volvulus* Mf within six months following IVM treatment, while comparing the performance of 3 independent diagnostic techniques: microscopy, actin-qPCR and O-150 LAMP.

## Method

### Study area

**Fig. 1:**
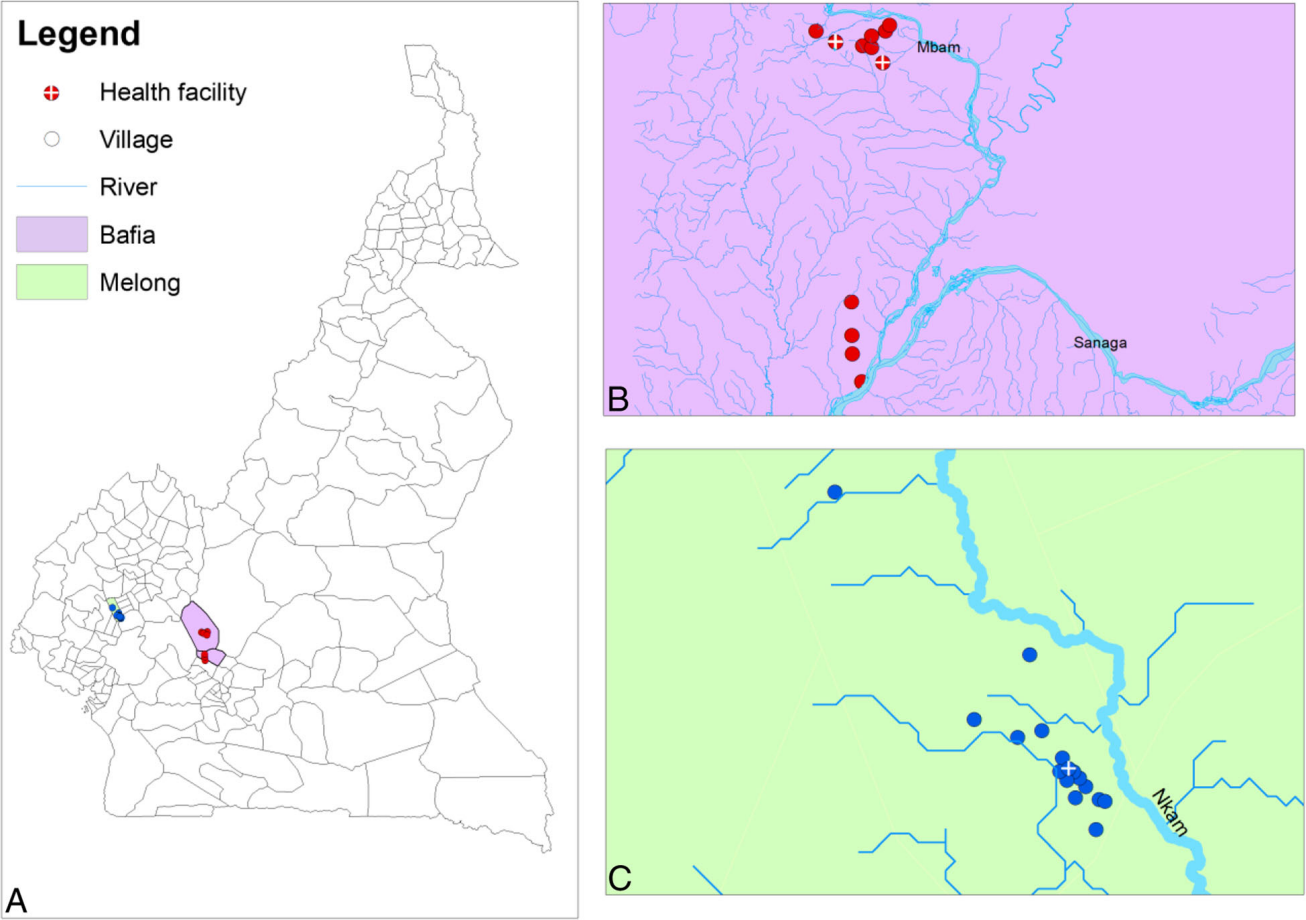
Overview of the study area including Bafia and Melong Health Districts and study communities. This map was created using ArcGIS (ArcMap v10.5.1) software

This study was carried out in the Bafia (more than 20 rounds of annual IVM-MDA, with forest-savannah transitional ecosystem) and Melong (10 rounds of annual IVM-MDA, with forest bioecology) Health Districts (HD) situated in the Centre and Littoral Regions of Cameroon, respectively (Fig. 1a). These areas were among the onchocerciasis foci that benefited from APOC oriented CDTI program in Cameroon, but differ in the number of annual rounds of MDA. Twenty-three first-line (closest to the breeding sites) and second-line villages (5–10 km away from breeding sites) grouped into 14 communities were purposely selected for the study. First and second line communities were selected as their infection prevalence are usually higher due to the fact that they are closer to the vector breeding sites, hence higher biting rates and potentially higher infection rate in the human population. Villages that were very close to each other (< 2 km apart) were treated as belonging to the same community.

### Bafia Health District

In this (HD), nine villages along the Mbam River, grouped into six communities (Balamba 1, Balamba 2, Biamo, Botatango/Boalondo, Lable/Nyamsong and Ngomo/Biatsota) were purposely selected for the study (Fig. 1b). The Bafia HD is in the Mbam and Inoubou Division of the Centre Region of Cameroon. It belongs to the Centre 1 CDTI project area and has had over 20 rounds of annual CDTI but is still meso endemic for onchocerciasis [39]. The altitude of this forest savanna transition zone varies from 1100 to 1300 m above sea level and lies between coordinates 4°45′00″ north and 11°14′00″ east. The main activities of the inhabitants are agriculture (mainly cocoa and cassava production) along the river beds, fishing and sand mining, all of which expose them to repeated *Simulium* bites. The Mbam river offers an excellent breeding site for *Simulium* throughout the year because of the presence of Mape dam that releases water from its lake during the dry season to regularize the course of the Sanaga river in view of stabilizing the production of electrical energy at Edea. Most of the first-line communities in the Bafia HD are situated close to the main river.

### Melong Health District

In this HD, fourteen first and second-line villages along the Nkam River and its tributaries grouped into eight communities (Mounko, Manjibo, Singa/Mbie/Barembeng2/Longze, Ndoumbot/Ntangtom, Nkoniakoniama/Nkonianke/Nkoniambot, Ndom-Bakem, Barembeng1 and Mpaka) were purposely selected for the study (Fig. 1c). The Melong HD is in the Mungo Division of the Littoral Region of Cameroon and belongs to the Littoral 2 CDTI project areas. It has received 10 rounds of annual IVM-MDA with a drastic drop in disease prevalence (over 20% nodule prevalence in adult males before CDTI [40] which could be translated to approximately over 40% Mf prevalence as reported in areas naïve to ivermectin treatment [41], to the observed 11.7% microfilaridemia among participants screened for this study). The district is located in a forest ecosystem and the main activity of the inhabitants is farming of cocoa, coffee and palm oil. Villages in this HD are not too close to the main river Nkam and the transmission is more ensured by the tributaries of river Nkam which are affected negatively by the reduction in water volume during the dry season.

### Study design

**Fig. 2:**
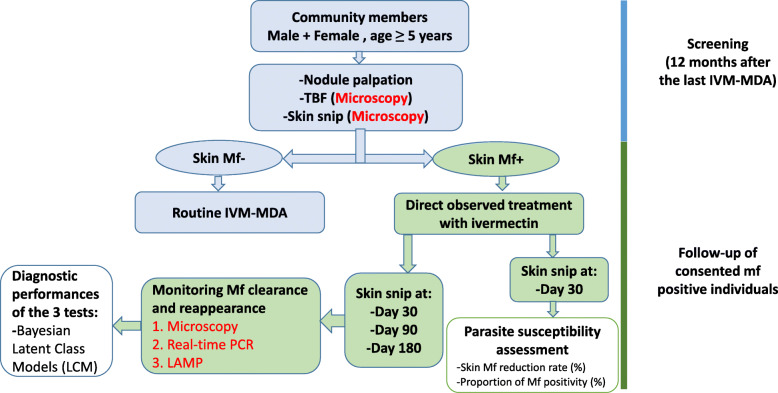
Overview of the study design

The study design is summarized in Fig. 2. The field activities spanned a period of eight months (May 2016 to January 2017) beginning with sensitization and mobilization of the populations. All members in the selected communities that met the eligibility criteria were invited to take part in the screening at the time when the research team arrived at each of them.

Convenience sampling was used to recruit eligible subjects to be included in the follow up study, whereby, residents of selected communities were screened for onchocerciasis at 12 months after the last IVM-MDA. Individuals aged 5 years and above who had lived in the community for at least 5 years and consented/assented to participate in the study were examined for the presence of palpable nodules and Mf in the skin (*O. volvulus*) while *Mansonella perstans* and *Loa loa* infections were determined microscopically using thick blood film (TBF). No night blood for detection of *Wuchereria bancrofti* Mf was performed since recently it has been shown that the parasite is not endemic in this area of Cameroon [42]. Individuals who were not microfilaridermic following skin snip examination, underwent IVM-MDA using the routine CDTI strategy. Consenting *O. volvulus* microfilaridermic individuals who were negative for *M. perstans and L. loa* were treated with IVM under direct observation (DOT) by health personnel who were members of the research team. All skin snip positive individuals who did not take treatment under direct observation and those who did not sign an informed consent for the follow-up study were excluded. The dosage of IVM was calculated according to the individual’s height [43]. All persons that were not recruited for this study were encouraged to take part in the routine annual MDA.

Following DOT with ivermectin under the supervision of a health worker who was a member of the research team, skin snips were collected after 1 month (D30) and examined for *O. volvulus* Mf. Susceptibility to IVM 1 month after treatment was determined using two approaches: (i) determining percent reduction of Mf by comparing the geometric mean density of Mf per skin snip (GMMfD/ss) at D30 to pre-treatment levels and (ii) determining the proportion of microfilaridemia positivity. In addition, skin Mf was monitored at 1 month (D30), 3 months (D90) and 6 months (D180) using microscopy, actin-qPCR and O-150 LAMP techniques. By identifying eligible participants using a less sensitive method (microscopy) and monitoring the outcome of IVM treatment at 30-, 90- and 180-days using microscopy actin-qPCR and O-150 LAMP, this study design intended to circumvent the use of microscopy (an imperfect gold standard) for comparison. The Bayesian Latent Class model (described below) was used to compute the performance characteristics of the three diagnostic tests.

### Nodule palpation

Nodule palpation was carried out as described by Wanji and colleagues [44]. Following their consent, participants were partially undressed and examined using Rapid Epidemiological Assessment (REA) guidelines [45–47] with emphasis being made to the bony prominences of the iliac crest, torso, knees, arms, head,upper trochanter and femur. The number of nodules found were recorded and their positions noted on anatomical diagrams on the participant recruitment forms.

### Preparation and microscopy of thick blood film (TBF)

Day Blood (50 μL) was collected from 8 AM to 4 PM with a non-heparinized microcapillary tube (soda lime glass, Modulohm A/S Herlev, Denmark.) to identify *Loa loa* or *Mansonella perstans* infections**.** The microcapillary tube was centered on a clean slide then the blood was smeared in a circular area of about 1.5 cm. The smears were then air dried, packaged for transport back to the base and finally stained with 10% Giemsa within 24 h. The stained smears were examined using a light microscope (Brunel Microscopes Ltd., Chippenham, United Kingdom) with 10× (or 40×) objective lens for blood dwelling Mf. Any Mf present, were identified, quantified and recorded.

### Collection and microscopy of skin biopsies

Skin snipping was carried out as previously described [44]. In summary, two bloodless skin biopsies were taken, one from each iliac crest using a 2 mm corneo-scleral punch (CT 016 Eberhard’s 2218–15 C, Germany). The snipped areas were dusted with Baneocin antiseptic powder. The skin samples from each participant were placed in two separate wells of a 96 well microtiter plate containing 100 μL of saline. The plates were then sealed with parafilm to prevent any spill-over or evaporation and were incubated at room temperature for 24 h. Emerged microfilariae were counted using a light microscope at 10x magnification and each result was expressed per skin snip [44, 48]. Then, residual skin biopsies were transferred into 1.5 mL tubes (Eppendorf AG, Hamburg, Germany) containing 80% ethanol (GAPUMA UK Limited) and stored at − 20 °C for DNA extraction and PCR and LAMP analyses.

### Genomic DNA extraction from skin biopsies

Skin biopsies were thawed and placed into 2 mL tubes (Eppendorf AG) containing 160 μL of 1X Phosphate Buffered Saline (SIGMA-ALDRICH, USA) and 18–20 (1.0–1.3 mm) glass beads (VWR International, Darmstadt, Germany) to be homogenized at 7000 rpm for 180 s using the MagNA Lyser Instrument (Roche Diagnostics GmbH, Mannheim, Germany). Then, the QIAGEN DNeasy® Blood & Tissue Kit (250) (Qiagen, Hilden, Germany) was used to extract genomic DNA according to the manufacturer’s instructions. Briefly, 180 μL of ALT buffer and 20 μL of proteinase K were added into the homogenate and incubated at 56 °C in a water bath overnight. Following the overnight incubation, samples were vortexed for 15 s before addition of 200 μL of AL buffer and incubated for another 10 min at 56 °C. Then 200 μL of 96% ethanol (GAPUMA UK Limited) were added and mixed with the sample before pipetting into a DNeasy Mini spin column which was placed in a 2 mL collection tube. After centrifugation at 8000 rpm for 60 s, the flow through and collection tubes were discarded and the spin column was placed in a new 2 mL collection tube. Then, two washing steps using 500 μl of AW1and AW2 were performed before the DNA was eluted by adding 200 μL of the AE elution buffer. Upon 10 min incubation at room temperature the DNA was collected into a 1.5 mL tube (Eppendorf AG) through centrifugation for 60 s at 8000 rpm. Finally, the eluted DNA was stored at − 20 °C until use for actin-qPCR and LAMP assays.

### *O. volvulus-*specific duplex real-time PCR

We carried out specific real-time PCR (OvwFtsZ/actin duplex real-time PCR) to determine the presence of *O. volvulus* and/or *Wolbachia* endobacteria infection in skin biopsies. This PCR was performed using Qiagen’s QuantiNova Probe PCR Kit (Qiagen, Hilden, Germany) including Hotstar Taq, 10X buffer and 25 mM MgCl_2._ Prior to the assay, DNA samples from the skin biopsies were tested for PCR inhibitory factors using Mouse IFN-γ real-time PCR as described previously [28], but no inhibition was detected (S1 Fig pdf). The actin-2 gene (GenBank: M84916) for the *O. volvulus* nematode and wFtsZ gene (GenBank: AJ276501) for *Wolbachia* endobacteria were used. The PCR was conducted using 2 μL DNA in 10 μL volume in a RotorGene 6000 (Qiagen) with the following reaction mixture: 1X QuantiNova Probe PCR Master Mix (Qiagen), 0.4 μM OvwFtsZ Fw (5′-AGGAATGGGTGGTGGTACTG-3′), 0.4 μM OvwFtsZ Rev. (5′-CTTTAACCGCAGCTCTTGCT-3′), 0.4 μM OvActin Fw (5′-GTGCTACGTTGCTTTGGACT-3′), 0.4 μM OvActin Rev. (5′-GTAATCACTTGGCCATCAGG-3′), 0.025 μM OvwFtsZ TaqMan Probe (Fam-CCTTGCCGCTTTCGCAATCAC-3′), 0.05 μM OvActin TaqMan Probe (JOE-AACAGGAAATGGCAACTGCTGC-3′). The cycling conditions were 95 °C for 2 min and 45 cycles of 95 °C 5 s and 58 °C 30 s. Fluorescence was acquired on the FAM (Green) and JOE (Yellow) channels at the end of the hybridization step. Plasmids (E^6^ copies/μL) containing the respective sequences were used as PCR positive controls in every run. Signals were analyzed using Rotor-Gene Software version 2.3.1 with threshold set to 0.02 and an outlier removal of 15%. A positive signal (maximum C_t_ value of 33) in the OvActin PCR was considered positive for *O. volvulus* infection. We standardized the experiment by running a 1:10 serial dilution of the plasmid DNA. The real-time PCR had a limit of detection of 10 copies/μL, meaning that 2/3 replicates had the same C_t_ (S2 Fig pdf). For this study, only the OvActin results were considered in determining *O. volvulus* Mf in the skin snips.

### *O. volvulus*-specific colorimetric LAMP assay

To detect *O. volvulus* DNA from skin biopsies, we performed the colorimetric O-150 LAMP assay as previously described [22] with some modifications. The primer sets consisted of the following sequences; **F3**: Forward outer primer (5′-TGGAAATTCACCAAAATATGGT-3′), **B3**: Backward outer primer (5′-GGGTACGTACCTTCAAACTG-3′), **FIP**: Forward inner primer (5′-TGATGACCTATGACCCTAATCTCAACGAATATTTTTCTTAGGACCCAAT-3′), and **BIP**: Backward outer primer (5′-TGAAAATGCGTTTTTCGCCGGGGTCCTAAGAAAAATATTCGACTA-3′). LAMP reactions contained 1.6 μM each of primers FIP and BIP, 0.2 μM each of F3 and B3, in 1X WarmStart Colorimetric LAMP Master Mix (New England Biolabs Inc., Ipswich, USA) with 2 μL of template DNA, or H_2_0 for non-template controls (NTCs) in a total volume of 25 μL. Reactions were incubated at the optimal temperature of 64 °C for 60 min in a GeneAmp®, PCR System 9700 (Applied Biosystems, Foster City, USA). Amplification resulted in a color change from pink to yellow in positive samples, while negative samples remained pink with no ambiguity in color determination when read against a white background (white A4 paper). Because of the high sensitivity of LAMP, DNA contamination and carry over of amplified products was prevented by using filter tips at all times, cleaning all work surfaces with 10% bleach solution before and after each session of work, performing each step of the analyses in separate work areas and minimizing manipulation of the reaction tubes. The assay was optimized using DNA from *O. volvulus* microfilariae as positive control and nuclease free water as negative control. DNA samples from *M. perstans* and *L. loa* were also used to confirm the specificity of the test.

### Data management and analyses

Data collected were recorded into a template developed in Microsoft Excel 2013 and later exported to SPSS version 20 (IBM SPSS Statistics 22; Armonk, NY) for statistical analysis. All differences were considered statistically significant at *P*-values < 0.05. Proportion of Mf positivity was expressed as a percentage of the number examined at different time points of the follow ups. Chi-square test was used to check for significant differences in the positive rates between the screening techniques at different screening time points.

A web-based application described by Lim et al. [49] and based on Bayesian Latent Class Models (LCM) was used to determine the accuracy (sensitivity, specificity, positive predictive value (PPV) and negative predictive value (NPV)) of the diagnostic tests using microscopy as an imperfect gold standard with the help of a simplified interface of three-tests in one-population model (Walter and Irwig model) [49]. In brief, Bayesian LCMs estimate accuracies of diagnostic tests based on the true disease status of each patient. Bayesian LCMs do not assume that any diagnostic test or combination of diagnostic tests is perfect [50, 51]. Table S1 shows the data input into the Web-based application template.

## Results

### Nodule prevalence

Nodule prevalence of 41.5% (161/388) and 28.4% (170/599) were observed in the Bafia and Melong HDs, respectively.

### Identification of mf positive participants eligible for direct IVM treatment and follow-up

In the Bafia HD (Table 1), a total of 388 participants composed of 311 adults (≥20 years) and 77 children (5–19 years) were recruited for screening by microscopy. Most of the adults are farmers (236 participants). The gender and age group distribution in each community were determined (Table 1, S2 Table). Of 388 individuals who provided skin biopsies (Table 1), 105 (27.1%) were positive for skin Mf. The distribution of microfilaridemic positivity within the study communities were statistically significant (*P* = 0.001). The villages of Boalondo/Botatango had the highest level of endemicity (45.3%). Of the 105 individuals positive for skin Mf, 73 were males and 32 were females. Males (31.9%) had significantly higher infection rates (*P* = 0.018) than females (20.1%). A total of 30.4% (95/312) of adults were positive for skin Mf compared with 13.2% (10/76) of children and the difference between these 2 groups was statistically significant (*P* = 0.007).

**Table 1:** Screened participants positive for *O. volvulus* Mf in the Bafia HD

Variables	Study populations	Number examined	Number positive	Percentage (%)	***P*** – value (x^**2**^ test)
**Sex**	Male	229	73	31.9	0.018
	Female	159	32	20.1	
	**Total**	**388**	**105**		
**Age- group**	Children (5–19 years)	76	10	13.2	0.007
	Adults (≥20 years)	312	95	30.4	
	**Total**	**388**	**105**	**27.1**	
**Communities**	Balamba 1	28	7	25.0	0.001
	Balamba 2	57	5	8.8	
	Biamo	68	23	33.8	
	Boalondo/Botatango	53	24	45.3	
	Lable/Nyamsong	88	12	13.6	
	Ngomo/Biatsota	94	34	36.2	
	**Total**	**388**	**105**	**27.1**	

In the Melong HD (Table 2), the 599 participants comprised 467 adults, who were mainly farmers, and 132 children. The gender and age group distribution in each community were determined (S3 Table).

**Table 2:** Screened participants positive for *O. volvulus* Mf in the Melong HD

Variables	Study populations	Number examined	Number positive	Percentage (%)	***p*** – value (x^**2**^ test)
**Gender**	Male	283	44	15.5	0.005
	Female	316	26	8.2	
	**Total**	**599**	**70**	**11.7**	
**Age group**	Children (5–19 years)	132	15	11.4	0.896
	Adults (≥20 years) (	467	55	11.8	
	**Total**	**599**	**70**	**11.7**	
**Community**	Mounko	60	4	6.7	0.002
	Manjibo	51	12	23.5	
	Singa/Mbie/Barembeng2/Longze	104	10	9.6	
	Ndoumbot/Ntangtom	55	14	25.5	
	Nkoniakoniama/Nkonianke/Nkoniambot	87	7	8.0	
	Ndom-Bakem	79	10	12.7	
	Barembeng1	101	8	7.9	
	Mpaka	62	5	8.1	
	**Total**	**599**	**70**	**11.7**	

Following evaluation (Table 2), 70 (11.7%) participants were found positive for skin Mf. The villages of Ndoumbot/Ntangtom in the Melong HD had the highest proportion of infected persons (25.5%). Of the 70 individuals positive for skin Mf, 44 (15.5%) were males and 26 (8.2%) were females, and this difference was statistically significant (*P* = 0.005). Overall, 11.8% (55/467) of adults and 11.4% (15/132) of children were positive for Mf. Thus, the Bafia HD had significantly higher skin Mf proportions compared to the Melong HD (*P* = 0.002).

From the 987 persons screened in the Bafia and Melong HDs, 175 microfilaridemic individuals were identified as potential candidates for the follow-up study. However, follow-up samples were only available from those who voluntarily consented.

In total 51 and 44 individuals could be recruited for the follow up study from the Bafia and Melong HD, respectively. The socio-demographic data from those individuals are shown in S4 and S5 Tables. Comparison analyses were made only for eligible participants (51, 51 and 48 at 1-, 3- and 6-months post-treatment in the Bafia HD as well as 29, 44 and 38 at 1, 3 and 6 months after treatment in the Melong HD) from whom samples were collected at each of follow-up time points. A total of 150 skin snip samples from Bafia HD and 111 skin snip samples from Melong HD were collected for analysis (S6 table). None of the microfilaridemic participants were co-infected with *M. perstans* or *L. loa* as determined by thick blood films.

### Susceptibility of *O. volvulus* microfilariae to ivermectin in two areas of contrasting MDA history following direct observed IVM treatment

**Fig. 3:**
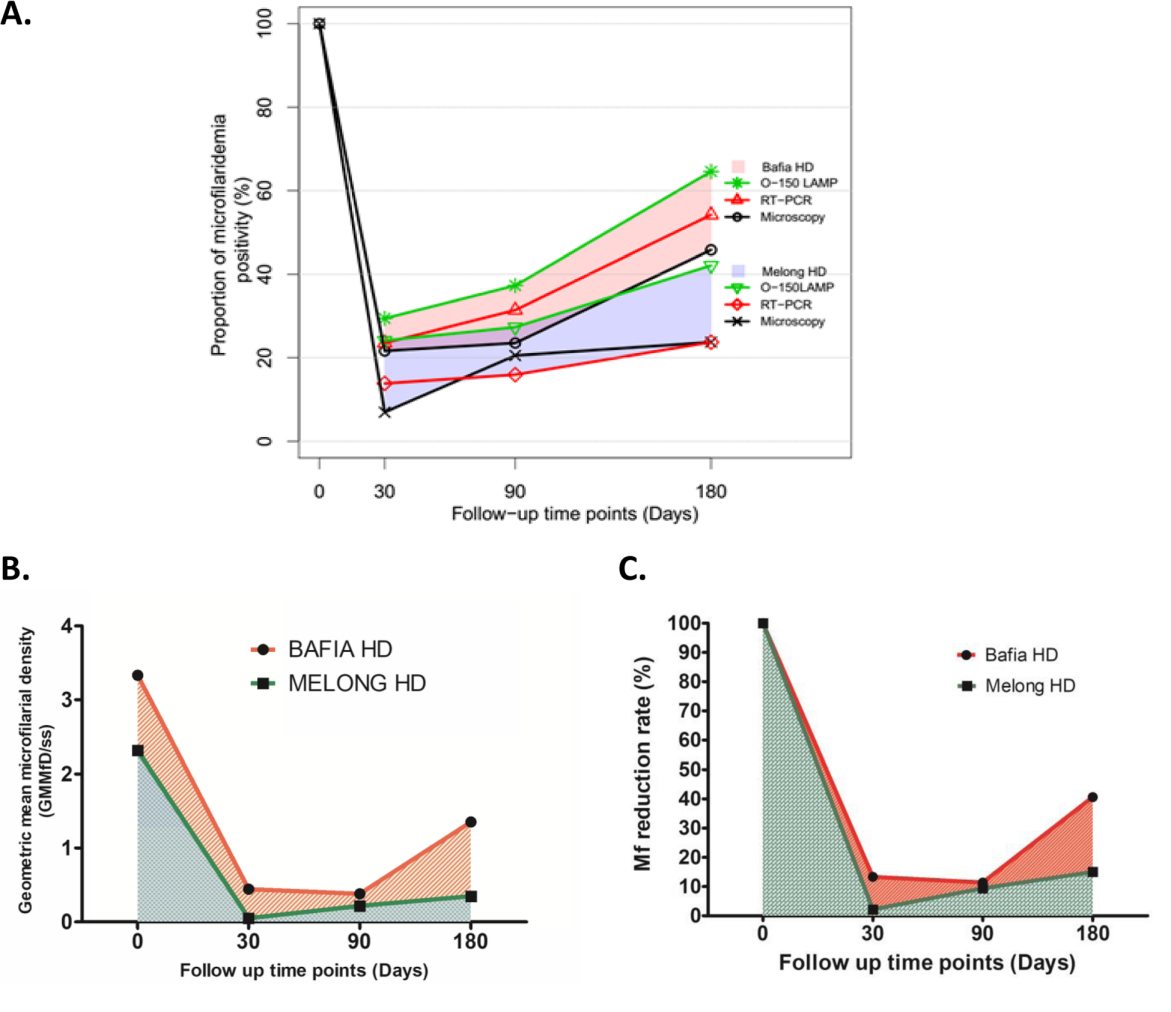
Microfilaridemia clearance and repopulation dynamics at 30, 60 and 90 days post direct observed IVM treatment in the Bafia and Melong HDs: **a** Proportion of microfilaridemia positivity. **b** Geometric mean microfilarial density per skin snip (GMMfD/ss). C) Percent reduction in GMMfD/ss

Ivermectin led to a rapid drop in the proportion of microfilaridemic individuals after one month of treatment in both study sites (Fig. 3a). The proportion of participants detected with infection at the 1-month follow-up was significantly higher in the Bafia HD (21.6%) compared to the Melong HD (6.9%). The repopulation trend showed an increase in proportions of microfilaridemic individuals over time (D30 prevalence < D90 prevalence < D180 prevalence) by all techniques. However, the repopulation rate was faster in Bafia than Melong HD, presented by the larger surface area under the curves for geometric mean Mf density (Fig. 3b) and Mf reduction rate (Fig. 3c).

In detail, the pre-treatment geometric mean density for the 51 individuals recruited for follow-up in Bafia HD was 3.331 Mf/ss. At 1-month follow-up, the geometric mean Mf density per skin snip (GMMfD/ss) was 0.442 Mf/ss. When the 1-month GMMfD/ss was compared to the pre-treatment level, we observed 13.3% of the initial density (86.7% Mf reduction rate) in the geometric mean skin Mf (Fig. 3c). IVM treatment elicited a statistically significant difference in the geometric mean density of skin microfilaridemia after 30 days (*P* < 0.001). At day 90 post treatment, all 51 follow-up participants were again examined and the GMMfD/ss at this time point was 0.379 Mf/ss. When compared to the pre-treatment level of 3.331 Mf/ss, we had 11.4% of the initial density left which resulted to 88.6% Mf reduction rate. After six months, 48 participants were examined for the fourth and last time of the study and the GMMfD/ss was 1.353 Mf/ss. The Mf reduction rate at 6 months was 59.4% with a skin microfilaria repopulation rate of 40.6%. The reduction in geometric mean density of skin microfilaridemia after 6 months was still significant (*P* = 0.01). The trend in geometric mean Mf reduction rate at different follow-up time points and repopulation of skin Mf shows a sharp decrease (86.7%) within 1 month of treatment, a further but slower decrease to 88.6% between one and three months and a rapid repopulation rate of 40.6% between the third and the sixth months after treatment (Fig. 3b).

In the Melong HD pre-treatment geometric mean density for the 44 individuals recruited for follow-up was 2.318 Mf/ss. At 1-month follow-up, it decreased to 0.051 for the 29 participants examined giving a reduction rate of 97.8% (Fig. 3c). Repopulation gradually sets in at the 3-month follow-up at a rate of 9.4%, but remained low even after 6 months (15%). The trend in geometric mean Mf reduction rate at different follow-up time points and repopulation of skin Mf (Fig. 3b) shows a sharp decrease (97.8%) within 1 month of treatment, and a very slow repopulation rate from three to sixth months after treatment (Fig. 3b and c).

### Monitoring skin mf rates after IVM treatment using microscopy, PCR and LAMP technologies

In the Bafia HD, 51 eligible individuals were present for the follow-ups and a total of 150 skin snips were collected (Table 3).

**Table 3:** Microfilaridemia detection rates by microscopy, actin-qPCR and O-150 LAMP at day 30, 60 and 180 post IVM treatment in Bafia HD

Time point after treatment (days)	Number of consented eligible participants	Number of samples collected	Proportion positive for microscopy n(%)	Proportion positive for real-time PCR n(%)	Proportion positive for O-150 LAMP n(%)
**D30**	51	51	11 (21.6%)	12 (23.5%)	15 (29.4%)
**D90**	51	51	12 (23.5%)	16 (31.4%)	19 (37.3%)
**D180**	51	48	22 (45.8%)	26 (54.2%)	31 (64.6%)
**Total**		**150**	**45 (30%)**	**54 (36.0%)**	**65 (43.3%)**
**Significance**			P = 0.014	P = 0.005	P = 0.001

n = number of cases detected by each method

Proportion of microfilaridemia positivity detected by microscopy were 21.6% (11/51) after one month, 23.5% (12/51) after three months, and 45.8% (22/48) after six months. There was a statistically significant difference (*P* = 0.014) in the prevalence levels obtained at the different times of screening.

For the real-time actin-qPCR, a sample was considered positive when the actin signal (in duplicate) was above the threshold level. A representative set of samples from individuals are shown in S3 Fig. This method detected microfilaridemia proportion positivity of 23.5% (12/51), 31.4% (16/51) and 54.2% (26/48) at 1-, 3- and 6-months post treatment, respectively. There was a significant difference in the real-time actin-qPCR prevalence at the different time points (*P* = 0.005).

A colorimetric O-150 LAMP assay with a simple visual readout (S4 Fig) was also used to detect infection in DNA extracted from skin snips. Prior to initiation of the amplification reaction, samples were pink. After a 60 min incubation at 64 °C, samples turned yellow in the presence of *O. volvulus* DNA (S4A Fig). Samples remained pink in the absence of template, or if DNA from *M. perstans* or *L. loa* was present (S4B Fig), confirming the specificity of the LAMP assay. Colorimetric LAMP detected infection in 15/51 individuals (29.4%) at 1-month following chemotherapy, while 19/51 (37.3%) and 31/48 (64.6%) were detected after 3- and 6-months, respectively. A significant difference in the proportion of skin microfilaridemia positivity was also observed between the follow-up time points when using LAMP to detect infection (*P* = 0.001).

**Fig. 4:**
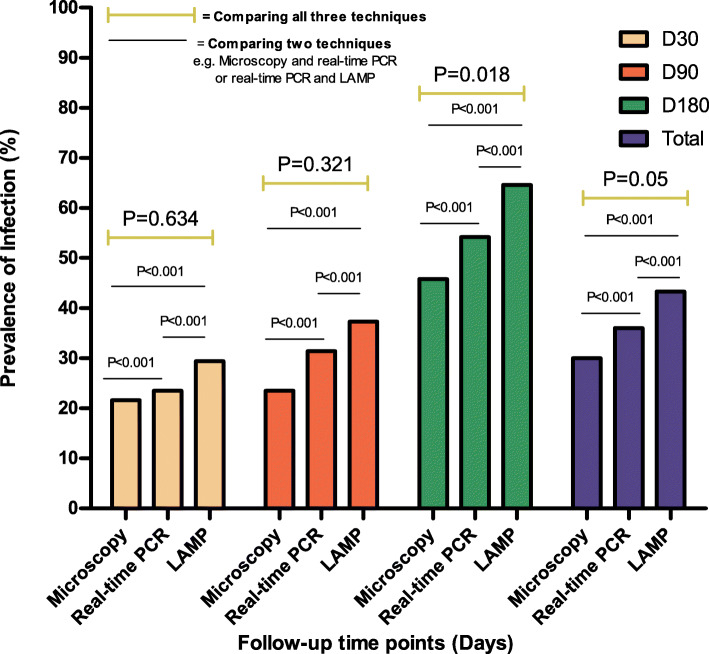
*O. volvulus* microfilaridermia positivity rate at different time points following IVM treatment in the Bafia health district using microscopy, actin-qPCR and O-150 LAMP assays

Despite the difference in targets for determining parasite infection, in Bafia HD, microscopy, actin-qPCR and LAMP showed the same trend post treatment, namely a steady increase in the proportion of microfilaridermia positivity 1-, 3- and 6-months post treatment (Fig. 4). The molecular assays were found to be more sensitive than microscopy, with highest levels of sensitivity obtained using LAMP. At each time point, the results obtained by comparing the different diagnostic methods were consistent (Microscopy < actin-qPCR < LAMP) though not always significantly different (Fig. 4). However, significant differences were seen when comparing two techniques (in pairs) at each time point (Fig. 4).

In the Melong HD, 44 eligible recruited individuals were present for the follow-up study and a total of 111 skin snip samples (Table 4) were collected from them at 1 month (29), 3 months (44) and 6 months (38) post direct observed treatment, respectively.

**Table 4:** Microfilaridemia detection rate by microscopy, actin-qPCR and O-150 LAMP at day 30, 60 and 180 post IVM treatment in Melong HD

Time point after treatment (days)	Number of consented eligible participants	Number of samples collected	Proportion positive for microscopy n(%)	Proportion positive for real-time PCR n(%)	Proportion positive for O-150 LAMP n(%)
**D30**	44	29	2 (6.9%)	4 (13.8%)	7 (24.1%)
**D90**	44	44	9 (20.5%)	7 (15.9%)	12 (27.3%)
**D180**	44	38	9 (23.7%)	9 (23.7%)	16 (42.1%)
**Total**		111	**20 (18.0%)**	**20 (18.0%)**	**35 (31.5%)**
**Difference**			*P* = 0.180	*P* = 0.520	*P* = 0. 215

n = number of cases detected by each method

**Fig. 5:**
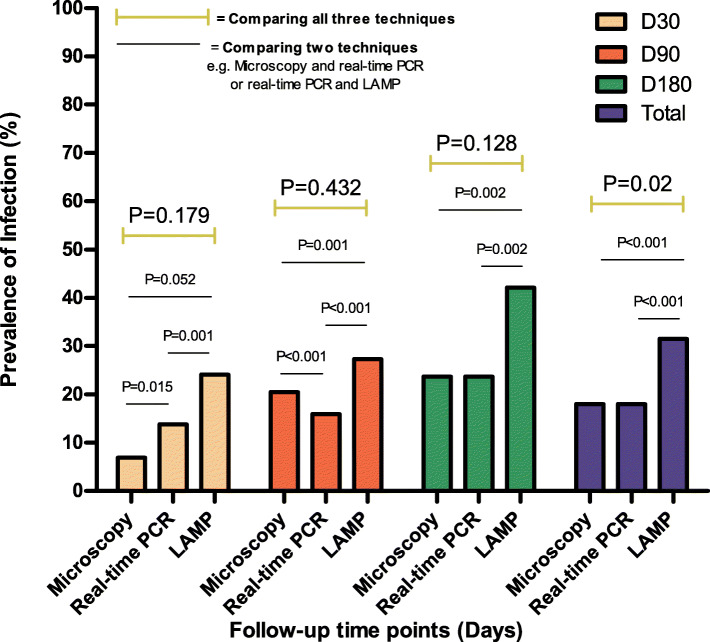
*O. volvulus* microfilaridemia positivity rate at different time points following IVM treatment in Melong HD using microscopy, actin-qPCR and O-150 LAMP assay

Microscopy detected proportions of Mf positivity of 6.9% (2/29) after one month, 20.5% (9/44) after three months and 23.7% (9/38) after six months. There was no significant difference in the positive proportions by microscopy at the different follow-up time points (*P* = 0.18), however the positivity increased with time from 1 to 6 months post treatment. Actin-qPCR detected 4/29 (13.8%), 7/44 (15.9%) and 9/38 (23.7%) at 1-, 3- and 6-months post treatment, respectively. Colorimetric LAMP detected infections in 7 of the 29 individuals (24.1%), 12/44 (27.3%) and 16/38 (42.1%) after 1-, 3- and 6-months post treatment, respectively. No significant difference was observed in the positive proportions between the follow-up times regardless of the methods (Fig. 5). However, after combining the samples collected at all points of monitoring (111), using the Chi square test, a significant difference (*P* = 0.02) was observed between the results from the three diagnostic methods. When comparing the performance of the techniques in pairs (e.g. Microscope vs actin-qPCR or LAMP vs actin-qPCR) significant differences in detecting infection where observed (Fig. 5). Here too, LAMP assay detected the highest microfilaridemia proportions compared to the other methods at each time point (Fig. 5).

An overview of the individual results of the three different methods and time points are shown in S6 table.

### Performance characteristics of actin-qPCR and O-150 LAMP assay using microscopy as the imperfect gold standard

The observed frequencies of various test result combinations were determined and entered into the data input template (S1 Table) of the Web-based application for each follow-up time point (D30, D90 and D180). The 3x positive Serial Number 1 (SN1) and 3x negative (SN8) have the highest numbers showing that actin-qPCR and O-150 LAMP confirm microscopy values. In addition, only positive in LAMP assay have the third highest observed frequency suggesting that the LAMP assay has a higher sensitivity compared to microscopy and actin-qPCR.

Finally, there are no observed frequencies when the LAMP assay is negative whereas microscopy and actin-qPCR are positive (SN2), further confirming the high sensitivity of LAMP.

The accuracy (sensitivity, specificity, positive predictive value (PPV) and negative predictive value (NPV)) of actin-qPCR and O-150 LAMP using microscopy as imperfect gold standard for skin biopsy samples collected from Bafia and Melong HDs are summarized in Tables 5 and 6, respectively.

**Table 5:** Performance characteristics of the three tests in Bafia HD. Summary of sensitivity, specificity, PPV and NPV for microscopy, actin-qPCR and O-150 LAMP at different time points following direct observed treatment of microfilaridermic individuals with ivermectin in Bafia health district

METHODS	SENSITIVITY	SPECIFICITY	PPV	NPV
**D30**				
MICROSCOPY	89.6 (61.6–99.9)	99.3 (93.0–100)	97.6 (76.9–100)	96.9 (86.2–100)
REAL-TIME PCR	89.6 (64.5–99.6)	96.8 (87.5–99.9)	89.6 (62.2–99.7)	96.8 (87.2–99.9)
LAMP ASSAY	97.6 (78.7–100)	92.1 (80.6–98.8)	79.0 (53.2–97.1)	99.2 (91.9–100)
**D90**				
MICROSCOPY	71.4 (45.8–91.8)	96.9 (88.0–99.8)	90.6 (67.0–99.5)	88.8 (74.8–97.3)
REAL-TIME PCR	97.5 (75.8–100)	97.0 (86.3–100)	93.2 (69.3–99.9)	98.9 (87.8–100)
LAMP ASSAY	97.5 (79.0–100)	88.7 (75.0–97.6)	78.6 (54.5–95.7)	98.8 (89.1–100)
**D180**				
MICROSCOPY	80.6 (62.9–93.1)	94.8 (81.3–99.6)	94.9 (80.6–99.6)	80.8 (61.5–93.4)
REAL-TIME PCR	98.9 (87.3–100)	98.5 (84.6–100)	98.8 (86.4–100)	98.7 (83.4–100)
LAMP ASSAY	99.0 (90.1–100)	76.6 (56.3–91.6)	83.4 (66.9–94.3)	98.6 (85.2–100)
**D30 + D90 + D180**				
MICROSCOPY	80.7 (68.4–90.2)	97.4 (93.1–99.5)	94.5 (85.1–98.9)	90.3 (83.1–95.2)
REAL-TIME PCR	98.1 (90.0–100)	97.6 (92.7–99.8)	95.7 (87.1–99.6)	99.0 (94.2–100)
LAMP ASSAY	99.4 (93.4–100)	87.1 (79.4–93.1)	80.7 (69.6–89.9)	99.6 (95.8–100)

**Table 6:** Performance characteristics of the three tests in Melong HD. Summary of sensitivity, specificity, PPV and NPV for Microscopy, actin-qPCR and O-150 LAMP at different time points following direct observed treatment of microfilaridermic individuals with ivermectin in Melong Health District

METHODS	SENSITIVITY	SPECIFICITY	PPV	NPV
**D30**				
MICROSCOPY	45.4 (10.1–91.2)	99.1 (89.7–100)	89.6 (29.3–100)	91.1 (71.0–99.2)
REAL-TIME PCR	87.6 (32.2–100)	98.7 (87.1–100)	92.2 (36.2–100)	98.0 (78.3–100)
LAMP ASSAY	94.2 (52.0–100)	89.3 (71.5–99.7)	60.5 (18.4–99.3)	98.9 (87.4–100)
**D90**				
MICROSCOPY	90.6 (48.3–100)	94.7 (82.3–100)	78.9 (35.2–99.9)	98.0 (82.0–100)
REAL-TIME PCR	38.0 (10.9–80.6)	99.4 (92.9–100)	93.1 (43.4–100)	87.8 (69.8–97.9)
LAMP ASSAY	92.1 (55.3–100)	89.8 (75.9–99.7)	66.1 (28.1–99.2)	98.1 (84.9–100)
**D180**				
MICROSCOPY	88.9 (58.1–99.9)	96.1 (84.9–99.8)	87.5 (59.2–99.4)	96.5 (84.5–100)
REAL-TIME PCR	96.7 (69.9–100)	98.8 (89.2–100)	96.2 (66.7–100)	99.0 (89.4–100)
LAMP ASSAY	97.1 (74.0–100)	75.3 (58.7–88.5)	55.0 (30.6–78.2)	98.9 (87.7–100)
**D30 + D90 + D180**				
MICROSCOPY	80.2 (57.2–95.0)	95.9 (89.4–99.2)	81.5 (56.6–96.4)	95.7 (87.9–99.0)
REAL-TIME PCR	78.3 (53.3–97.9)	99.7 (96.5–100)	98.2 (82.1–100)	95.4 (87.4–99.7)
LAMP ASSAY	98.5 (84.3–100)	84.7 (75.6–92.3)	58.7 (39.1–79.4)	99.6 (95.4–100)

The sensitivity of O-150 LAMP was generally higher than that of actin-qPCR in detecting *O. volvulus* infection following treatment with a microfilaricide (IVM) in both study sites (Tables 5 and 6) at different time points. Bafia HD recorded sensitivities of 99.4% for O-150 LAMP, 98.1% for actin-qPCR and 80.7% for microscopy while Melong HD had 98.5% for LAMP assay, 78.3% for actin-qPCR and 80.2% for microscopy. With the exception at 3 months follow up in Melong HD, molecular methods had higher and comparable sensitivities. In both study sites, microscopy and actin-qPCR techniques showed very high and comparable specificity values (always > 94%) while O-150 LAMP is relatively lower at all the follow-up time points (ranging between 75 and 92%). With exception at 1-month follow-up in Bafia HD, the actin-qPCR assay had the highest PPV of over 92% at the different time points. The LAMP had relatively lower PPV compared to actin-qPCR and microscopy. In terms of negative predictive value, LAMP had the highest values (ranging between 98.1–99.6%) while the NPV of microscopy and actin-qPCR were relatively lower and comparable.

## Discussion

For a microfilaricidal compound to be used in the control of onchocerciasis, the skin microfilarial clearance and repopulation after treatment must be monitored with the most appropriate, easy to use and available diagnostic tool. In this study, the susceptibility and repopulation of skin microfilariae of *O. volvulus* were monitored within six months of direct observed treatment with IVM in two areas with different bioecology and histories of MDA but with similar therapeutic coverage, using three diagnostic methods (microscopy, actin-qPCR and O-150 LAMP). Microscopy which is the diagnostic gold standard for the detection of infection in skin snips (though less sensitive when Mf density is very low) [11, 14], was used to identify study participants (microfilaridermic individuals) that were monitored for the outcome of direct observed treatment with ivermectin within 6 months using three independent detection tools (microscopy, actin-qPCR and O-150 LAMP). Before the direct observed treatment intervention (after 12 months of the previous routine annual MDA), it was observed that, despite long term IVM-MDA in both study sites, the proportion of infected individuals amongst those screened was still high (27.1%; ranging between 8.8–45.3% among communities in Bafia HD, and 11.7%; ranging between 7.9–25.5% among communities in Melong HD) and might indicate an apparent failure of MDA especially in Bafia. To determine the Mf clearance rate by ivermectin at 1-month post treatment of microfilaridemic individuals, the microscopy detected 21.6% (but with 84.6% reduction rate in geometric mean density of skin Mf) and 6.9% (but with 97.8% reduction rate in geometric mean density of skin Mf) in Bafia and Melong Health Districts, respectively. The higher proportion of positive participants at the 1-month follow-up (Fig. 3a) supported by the lower Mf reduction rate (Fig. 3b and c) could suggest the presence of suboptimal response to IVM or higher re-infection rates in Bafia compared to Melong Health District where we observed a lower proportion of positive participants after treatment and the Mf reduction rate was close to the expected 98–99% postulated by Basanez and colleagues [6] within 1–2 months after a single IVM dose. The higher percentages of Mf detected in Bafia at different time points (Fig. 3b) also shows that the parasite population in the area with over 20 years of annual MDA is less susceptible to IVM than those in Melong with a shorter exposure period (10 years). This finding corroborates previous documentation of suboptimal response of *O. volvulus* Mf to IVM in Cameroon and Ghana [7, 8]. Again, the skin Mf repopulation rate at the 6-month follow-up in Bafia (47,5%) is much higher than the expected repopulation rate of less than 20% of pre-treatment load for up to 10 months post treatment modelled for IVM in a naïve population of parasites [52]. This is another strong indication that worms with suboptimal response to IVM may be present in Bafia HD that have received over 20 rounds of IVM-MDA.

From the dynamics of detecting infection after treatment, all three diagnostic techniques used to monitor the outcome of intervention showed a steady and similar increasing trend in the proportion of microfilaridemia positive samples from D30 through D90 to D180 in both study sites (Figs. 4 and 5) despite their historical and ecological differences. This indicates that, all three techniques were able to follow skin Mf reappearance after treatment, but only differ in their level of sensitivity as the nucleic acid-based techniques detected more infections than microscopy and this is in agreement with earlier reports [12, 22, 23, 26, 36, 51, 53, 54].

As shown on Figs. 4 and 5, as well as Tables 5 and 6, the LAMP assay was the most sensitive of the three techniques in the detection of microfilaria in all the skin biopsies and at all monitoring time points in both study sites. This therefore implies that in terms of sensitivity, LAMP assay with its reported advantages over actin-qPCR in terms of cost, robustness and simplicity [55–59] could be the most appropriate diagnostic tool to detect the presence of *O volvulus* infection in humans [22, 23, 36] as well as monitor the clearance and reappearance of skin Mf following treatment with a microfilaricidal compound as observed in this study. However, we also observed that the specificity of LAMP is lower than the actin-qPCR and a very high specificity (≥99%) of a diagnostic technique is usually required for programme evaluation. As previously reported [12, 60], our results (Tables 5 and 6) also confirm the poor sensitivity of microscopy when Mf density is very low as it detected the fewest number of infections among the three techniques used for monitoring. It was also seen that, the sensitivity of microscopy in the Melong HD after one month of treatment was very low (45.4%) when compared to the 89.6% in the Bafia HD. These observations are in line with the reduction rates in geometric mean densities of skin Mf in both study sites (97.8 and 84.6% respectively). This could be due to the fact that the drug effect on the parasite population in Melong is more effective than on the parasite population in Bafia HD. Hence, treatment will lead to very low microfilaridermia in the Melong HD which will further affect the poor sensitivity of microscopy as reported earlier [11]. Surprisingly, we observed a few samples (2 in Bafia HD and 3 in Melong HD; S1 Table, SN5) that were positive for microscopy but negative for both nucleic acid-based tools and this could have compromised their specificity. This observation could be due to the fact that, this study used residual skin snip biopsies, which are biopsies after migration of the Mf outside of the tissue during the 24 h incubation. Thus, no *O. volvulus* DNA could be extracted from those skin biopsies. Alternatively, the microscopically assessed Mf could come from another filarial species (e.g. *Mansonella spp or L. loa*) as demonstrated in other studies [15, 16]. In addition to the high sensitivity, the specificity of LAMP assay is also high (ranging between 75 and 92%), though relatively lower than that of actin-qPCR and microscopy. All non-template control tubes as well as those with DNA from other filarial species (*M. perstans, L. loa*) also confirmed the assay’s species-specificity as they always remained negative. The comparatively lower specificity of LAMP in this study, could have been due to its ability to detecting more infections than the imperfect gold standard. For any assay to be considered in the monitoring of transmission in elimination programmes, specificity should be high (> 99%). For the fact that O-150 can be amplified from multiple species of *Onchocerca*, and the use of primers alone will not be definitive for detecting *O. volvulus* in the vectors, the O-150 LAMP in this study can only be used to detect *Onchocerca* infection in the vectors without trying to differentiate the species.

## Conclusion

We identified microfilaridermic individuals from two health districts with different IVM-MDA histories and levels of endemicity, treated them with a microfilaricide (IVM) then followed their microfilaridemia dynamics at different time points over a period of 6 months using three diagnostic tools. The trends in microfilaridemia clearance and repopulation after treatment with IVM were established with parasites in Bafia HD that have been exposed to IVM for over 20 years presenting suboptimal response characters (lower than expected clearance and faster than expected repopulation rates) to IVM when compared to those from the Melong HD with just 10 years of exposure to the drug. The results also support the already known higher sensitivity of actin-qPCR over microscopy in detecting *O. volvulus* skin Mf at all monitoring time points. The sensitivity of the two nucleic acid-based techniques were comparable even though O-150 LAMP detected the highest number of infections at all monitoring time points. However, microscopy and actin-qPCR displayed better and comparable specificity than the O-150 LAMP assay. The high sensitivity of O-150 LAMP added to its robustness, simplicity, and relative cost effectiveness suggest that this diagnostic method should be given consideration as one of the reference diagnostic tools for monitoring Mf clearance/reappearance in clinical trials and control/elimination programs of onchocerciasis especially in areas with limited resources if the specificity is improved to or above 99%.

### Limitations

The nucleic acid-based techniques were only used as a qualitative method to detect the presence or absence of parasite, not to quantify. Hence, they can be best appreciated (especially the LAMP assay) in monitoring infection in foci that are nearing elimination criteria and clinical trials that aim at eliminating all parasites after a given period of treatment. In addition, the different techniques had different targets for detection of infection. Whole microfilariae for microscopy, and different DNA targets for the molecular methods (real-time Ov-actin PCR and O-150 LAMP), which makes the direct comparison of microscopy to real-time PCR and LAMP in regards to sensitivity and specificity difficult to assess. Also, gene copy numbers for O-150 are several logs higher than actin causing a critical difference between the actin-qPCR and the O-150 LAMP.

## Supplementary information

**Additional file 1 S1 Fig** Check for inhibitory factors in DNA.

**Additional file 2 S2 Fig** Detection limits of OvActin real-time PCR.

**Additional file 3 S3 Fig** Representative data for a 45-cycle reaction for the duplex real-time PCR assay showing positive (above threshold level indicated by the red horizontal line) and negative (below threshold level) signals.

**Additional file 4 S4 Fig.** A. Representative Data obtained from individual samples using a colorimetric LAMP assay. Samples containing *O. volvulus* microfilariae turned yellow (+) and were scored positive. Negative skin snips samples remained pink (−). B. Specificity of colorimetric LAMP assay. Reactions contained no template DNA (>) or DNA from *Onchocerca volvulus* (OV), *Mansonella perstans* (MP) or *L. loa* (LL).

**Additional file 5 S1 Table.** Data input for the simplified interface of three-tests in one-population model (Walter and Irwig model) in Bafia and Melong Health Districts.

**Additional file 6 S2 Table.** Socio-demographic characteristics and distribution of participants screened by microscopy in the Bafia Health District.

**Additional file 7 S3 Table.** Socio-demographic characteristics and distribution of participants screened by microscopy in the Melong health district.

**Additional file 8 S4 Table.** Socio-demographic characteristics and distribution of participants that volunteered for the follow up study in the Bafia health district.

**Additional file 9 S5 Table.** Socio-demographic characteristics and distribution of participants that volunteered for the follow up study in the Melong health district.

**Additional file 10 S6 Table.** Table showing Mf. count, Ct values for real-time PCR and time to LAMP for all samples investigated.

**Additional file 11 S1 File.** Diagnostic test results in Bafia Health District.

**Additional file 12 S2 File.** Diagnostic test results in Melong Health District.

## Data Availability

All data generated or analyzed during this study are included in this manuscript and its supplementary information files.

## Abbreviations

PCR: Polymerase Chain Reaction
LAMP: Loop mediated isothermal Amplification
IVM: Ivermectin
DOT: Direct Observed Treatment
MDA: Mass Drug Administration
CDTI: Community Directed Treatment with Ivermectin
HD: Health District
Mf: Microfilaria
ss: Skin Snip
GMMf: Geometric Mean Microfilaria
PPV: Positive Predictive Value
NPV: Negative Predictive Value
TN: True Negative
TP: True positive
FN: False Negative
FP: False Positive
CI: Confidence Interval
NEB: New England Biolabs
FIP: Forward Inner Primer
BIP: Backward Inner Primer
F3: Forward outer primer
B3: Backward outer primer

## References

[1] WHO: Uniting to Combat Neglected Tropical Diseases. River blindness (Onchocerciasis). 2020 WHO roadmap target: Elimination. https://unitingtocombatntds.org/ntds/onchocerciasis/. (Accessed 20 Apr 2020). In*.*; 2020.

[2] Adewole SOaA, S.K. (2009). Clinical manifestation of Onchocerciasis in Ise - Orun local government, Ekiti state. Nigeria Pakistan J Nutri.

[3] Edungbola LD, Watts SJ, Kayode OO (1987). Endemicity and striking manifestations of onchocerciasis in Shao, Kwara state, Nigeria. Afr J Med Med Sci.

[4] Katawa G, Layland LE, Debrah AY, von Horn C, Batsa L, Kwarteng A, Arriens S, Specht S, Hoerauf A, D WT (2015). Hyperreactive onchocerciasis is characterized by a combination of Th17-Th2 immune responses and reduced regulatory T cells. PLoS Negl Trop Dis.

[5] Njim T, Ngum JM, Aminde LN (2015). Cutaneous onchocerciasis in Dumbu, a pastoral area in the north-west region of Cameroon: diagnostic challenge and socio-economic implications. Pan African med J.

[6] Basanez MG, Pion SD, Boakes E, Filipe JA, Churcher TS, Boussinesq M (2008). Effect of single-dose ivermectin on Onchocerca volvulus: a systematic review and meta-analysis. Lancet Infect Dis.

[7] Osei-Atweneboana MY, Awadzi K, Attah SK, Boakye DA, Gyapong JO, Prichard RK (2011). Phenotypic evidence of emerging ivermectin resistance in Onchocerca volvulus. PLoS Negl Trop Dis.

[8] Pion SD, Nana-Djeunga HC, Kamgno J, Tendongfor N, Wanji S, Njiokou F, Prichard RK, Boussinesq M (2013). Dynamics of Onchocerca volvulus microfilarial densities after ivermectin treatment in an ivermectin-naive and a multiply treated population from Cameroon. PLoS Negl Trop Dis.

[9] Churcher TS, Pion SD, Osei-Atweneboana MY, Prichard RK, Awadzi K, Boussinesq M, Collins RC, Whitworth JA, Basanez MG (2009). Identifying sub-optimal responses to ivermectin in the treatment of river blindness. Proc Natl Acad Sci U S A.

[10] Kamga GR, Dissak-Delon FN, Nana-Djeunga HC, Biholong BD, Ghogomu SM, Souopgui J, Kamgno J, Robert A (2018). Audit of the community-directed treatment with ivermectin (CDTI) for onchocerciasis and factors associated with adherence in three regions of Cameroon. Parasit Vectors.

[11] Taylor HR, Munoz B, Keyvan-Larijani E, Greene BM (1989). Reliability of detection of microfilariae in skin snips in the diagnosis of onchocerciasis. Am J Trop Med Hyg.

[12] Thiele EA, Cama VA, Lakwo T, Mekasha S, Abanyie F, Sleshi M, Kebede A, Cantey PT (2016). Detection of Onchocerca volvulus in skin snips by microscopy and real-time polymerase chain reaction: implications for monitoring and evaluation activities. Am J Trop Med Hyg.

[13] Kale OO, Bammeke AO, Ayeni O (1974). An evaluation of skin snip techniques used in the quantitative assessment of microfilarial densities of Onchocerca volvulus. Bull World Health Organ.

[14] Duke BO (1962). A standard method of assessing microfilarial densities on onchocerciasis surveys. Bull World Health Organ.

[15] Ta TH, Moya L, Nguema J, Aparicio P, Miguel-Oteo M, Cenzual G, Canorea I, Lanza M, Benito A, Crainey JL (2018). Geographical distribution and species identification of human filariasis and onchocerciasis in Bioko Island, Equatorial Guinea. Acta Trop.

[16] Nana-Djeunga HC, Fossuo-Thotchum F, Pion SD, Chesnais CB, Kubofcik J, Mackenzie CD, Klion AD, Boussinesq M, Nutman TB, Kamgno J. Loa loa microfilariae in skin snips: consequences for onchocerciasis monitoring and evaluation in L. loa endemic areas. Clin Infect Dis. 2019;69(9):1628-1630. 10.1093/cid/ciz172 PMC6792118 30861060

[17] Basanez MG, Walker M, Turner HC, Coffeng LE, de Vlas SJ, Stolk WA (2016). River blindness: mathematical models for control and elimination. Adv Parasitol.

[18] Lobos E, Weiss N, Karam M, Taylor HR, Ottesen EA, Nutman TB (1991). An immunogenic Onchocerca volvulus antigen: a specific and early marker of infection. Science.

[19] Oguttu D, Byamukama E, Katholi CR, Habomugisha P, Nahabwe C, Ngabirano M, Hassan HK, Lakwo T, Katabarwa M, Richards FO (2014). Serosurveillance to monitor onchocerciasis elimination: the Ugandan experience. Am J Trop Med Hyg.

[20] Prince-Guerra JL, Cama VA, Wilson N, Thiele EA, Likwela J, Ndakala N, Muzinga Wa Muzinga J, Ayebazibwe N, Ndjakani YD, Pitchouna NA (2018). Comparison of PCR methods for Onchocerca volvulus detection in skin snip biopsies from the Tshopo Province, Democratic Republic of the Congo. Am J Trop Med Hyg.

[21] Zimmerman PA, Guderian RH, Aruajo E, Elson L, Phadke P, Kubofcik J, Nutman TB (1994). Polymerase chain reaction-based diagnosis of Onchocerca volvulus infection: improved detection of patients with onchocerciasis. J Infect Dis.

[22] Alhassan A, Osei-Atweneboana MY, Kyeremeh KF, Poole CB, Li Z, Tettevi E, Tanner NA, Carlow CK (2016). Comparison of a new visual isothermal nucleic acid amplification test with PCR and skin snip analysis for diagnosis of onchocerciasis in humans. Mol Biochem Parasitol.

[23] Lagatie O, Merino M, Batsa Debrah L, Debrah AY, Stuyver LJ (2016). An isothermal DNA amplification method for detection of Onchocerca volvulus infection in skin biopsies. Parasit Vectors.

[24] Williams JF, el Khalifa M, Mackenzie CD, Sisley B (1985). Antigens of Onchocerca volvulus. Rev Infect Dis.

[25] Toe L, Merriweather A, Unnasch TR (1994). DNA probe-based classification of Simulium damnosum s. l.-borne and human-derived filarial parasites in the onchocerciasis control program area. Am J Trop Med Hyg.

[26] Lloyd MM, Gilbert R, Taha NT, Weil GJ, Meite A, Kouakou IM, Fischer PU (2015). Conventional parasitology and DNA-based diagnostic methods for onchocerciasis elimination programmes. Acta Trop.

[27] Meredith SE, Lando G, Gbakima AA, Zimmerman PA, Unnasch TR (1991). Onchocerca volvulus: application of the polymerase chain reaction to identification and strain differentiation of the parasite. Exp Parasitol.

[28] Colebunders R, Mandro M, Mokili JL, Mucinya G, Mambandu G, Pfarr K, Reiter-Owona I, Hoerauf A, Tepage F, Levick B (2016). Risk factors for epilepsy in bas-Uele Province, Democratic Republic of the Congo: a case-control study. Int J Infect Dis.

[29] Gilbert J, Nfon CK, Makepeace BL, Njongmeta LM, Hastings IM, Pfarr KM, Renz A, Tanya VN, Trees AJ (2005). Antibiotic chemotherapy of onchocerciasis: in a bovine model, killing of adult parasites requires a sustained depletion of endosymbiotic bacteria (Wolbachia species). J Infect Dis.

[30] Osue HO, Inabo H, Yakubu S, Audu P, Mamman M (2018). Onchocercal DNA amplification using Beta actin gene primers compared with first internal transcribed spacer sequences for monitoring Onchocerciasis eradication strategy. Afr J Biomed Res.

[31] Francois P, Tangomo M, Hibbs J, Bonetti EJ, Boehme CC, Notomi T, Perkins MD, Schrenzel J (2011). Robustness of a loop-mediated isothermal amplification reaction for diagnostic applications. FEMS Immunol Med Microbiol.

[32] Kubota R, Vine BG, Alvarez AM, Jenkins DM (2008). Detection of Ralstonia solanacearum by loop-mediated isothermal amplification. Phytopathology.

[33] Tambo M, Mwinga M, Mumbengegwi DR (2018). Loop-mediated isothermal amplification (LAMP) and polymerase chain reaction (PCR) as quality assurance tools for rapid diagnostic test (RDT) malaria diagnosis in northern Namibia. PLoS One.

[34] Notomi T, Mori Y, Tomita N, Kanda H (2015). Loop-mediated isothermal amplification (LAMP): principle, features, and future prospects. J Microbiol.

[35] Lakwo T, Garms R, Wamani J, Tukahebwa EM, Byamukama E, Onapa AW, Tukesiga E, Katamanywa J, Begumisa S, Habomugisha P (2017). Interruption of the transmission of Onchocerca volvulus in the Kashoya-Kitomi focus, western Uganda by long-term ivermectin treatment and elimination of the vector Simulium neavei by larviciding. Acta Trop.

[36] Poole CB, Li Z, Alhassan A, Guelig D, Diesburg S, Tanner NA, Zhang Y, Evans TC, LaBarre P, Wanji S (2017). Colorimetric tests for diagnosis of filarial infection and vector surveillance using non-instrumented nucleic acid loop-mediated isothermal amplification (NINA-LAMP). PLoS One.

[37] Traore MO, Sarr MD, Badji A, Bissan Y, Diawara L, Doumbia K, Goita SF, Konate L, Mounkoro K, Seck AF (2012). Proof-of-principle of onchocerciasis elimination with ivermectin treatment in endemic foci in Africa: final results of a study in Mali and Senegal. PLoS Negl Trop Dis.

[38] Zarroug IM, Hashim K, ElMubark WA, Shumo ZA, Salih KA, ElNojomi NA, Awad HA, Aziz N, Katabarwa M, Hassan HK (2016). The first confirmed elimination of an Onchocerciasis focus in Africa: Abu Hamed, Sudan. Am J Trop Med Hyg.

[39] Kamga GR, Dissak-Delon FN, Nana-Djeunga HC, Biholong BD, Mbigha-Ghogomu S, Souopgui J, Zoure HG, Boussinesq M, Kamgno J, Robert A (2016). Still mesoendemic onchocerciasis in two Cameroonian community-directed treatment with ivermectin projects despite more than 15 years of mass treatment. Parasit Vectors.

[40] Wanji S, Tendongfor N, Nji T, Esum M, Che JN, Nkwescheu A, Alassa F, Kamnang G, Enyong PA, Taylor MJ (2009). Community-directed delivery of doxycycline for the treatment of onchocerciasis in areas of co-endemicity with loiasis in Cameroon. Parasit Vectors.

[41] Taylor HR, Duke BO, Munoz B (1992). The selection of communities for treatment of onchocerciasis with ivermectin. Trop Med Parasitol: official organ of Deutsche Tropenmedizinische Gesellschaft and of Deutsche Gesellschaft fur Technische Zusammenarbeit.

[42] Wanji S, Esum ME, Njouendou AJ, Mbeng AA, Chounna Ndongmo PW, Abong RA, Fru J, Fombad FF, Nchanji GT, Ngongeh G (2019). Mapping of lymphatic filariasis in loiasis areas: a new strategy shows no evidence for Wuchereria bancrofti endemicity in Cameroon. PLoS Negl Trop Dis.

[43] Alexander ND, Cousens SN, Yahaya H, Abiose A, Jones BR (1993). Ivermectin dose assessment without weighing scales. Bull World Health Organ.

[44] Wanji S, Kengne-Ouafo JA, Esum ME, Chounna PW, Tendongfor N, Adzemye BF, Eyong JE, Jato I, Datchoua-Poutcheu FR, Kah E (2015). Situation analysis of parasitological and entomological indices of onchocerciasis transmission in three drainage basins of the rain forest of south West Cameroon after a decade of ivermectin treatment. Parasit Vectors.

[45] Ngoumou P, Walsh JF, Mace JM (1994). A rapid mapping technique for the prevalence and distribution of onchocerciasis: a Cameroon case study. Ann Trop Med Parasitol.

[46] Noma M, Zoure HG, Tekle AH, Enyong PA, Nwoke BE, Remme JH (2014). The geographic distribution of onchocerciasis in the 20 participating countries of the African Programme for Onchocerciasis control: (1) priority areas for ivermectin treatment. Parasit Vectors.

[47] Zoure HG, Noma M, Tekle AH, Amazigo UV, Diggle PJ, Giorgi E, Remme JH (2014). The geographic distribution of onchocerciasis in the 20 participating countries of the African Programme for Onchocerciasis control: (2) pre-control endemicity levels and estimated number infected. Parasit Vectors.

[48] Schulz-Key H (1978). A simple technique to assess the total number of Onchocerca volvulus microfilariae in skin snips. Tropenmedizin und Parasitologie.

[49] Lim C, Wannapinij P, White L, Day NPJ, Cooper BS, Peacock SJ, Limmathurotsakul D. Using a Web-Based Application to Define the Accuracy of Diagnostic Tests When the Gold Standard Is Imperfect. PloS one. 2013. 10.1371/journal.pone.0079489. PMC3827152 24265775

[50] Limmathurotsakul D, Jamsen K, Arayawichanont A, Simpson JA, White LJ, Lee SJ, Wuthiekanun V, Chantratita N, Cheng A, Day NP (2010). Defining the true sensitivity of culture for the diagnosis of melioidosis using Bayesian latent class models. PLoS One.

[51] Trikalinos TA, Balion CM. Options for Summarizing Medical Test Performance in the Absence of a "Gold Standard". In: Chang SM, Matchar DB, Smetana GW, Umscheid CA, editors. *Methods Guide for Medical Test Reviews*. Rockville (MD): Oxford University Press; 2012.

[52] Basanez MG (2008). Mathematical modelling of parasitic infections: from data and parameter estimation to evolutionary implications. Preface Parasitol.

[53] Basanez MG, Boussinesq M (1999). Population biology of human onchocerciasis. Philos Trans R Soc Lond Ser B Biol Sci.

[54] Plaisier AP, van Oortmarssen GJ, Habbema JD, Remme J, Alley ES (1990). ONCHOSIM: a model and computer simulation program for the transmission and control of onchocerciasis. Comput Methods Prog Biomed.

[55] Alhassan A, Makepeace BL, LaCourse EJ, Osei-Atweneboana MY, Carlow CK (2014). A simple isothermal DNA amplification method to screen black flies for Onchocerca volvulus infection. PLoS One.

[56] Kaneko H, Kawana T, Fukushima E, Suzutani T (2007). Tolerance of loop-mediated isothermal amplification to a culture medium and biological substances. J Biochem Biophys Methods.

[57] Martzy R, Kolm C, Brunner K, Mach RL, Krska R, Sinkovec H, Sommer R, Farnleitner AH, Reischer GH (2017). A loop-mediated isothermal amplification (LAMP) assay for the rapid detection of Enterococcus spp. in water. Water Res.

[58] Notomi T, Okayama H, Masubuchi H, Yonekawa T, Watanabe K, Amino N, Hase T (2000). Loop-mediated isothermal amplification of DNA. Nucleic Acids Res.

[59] Takagi H, Itoh M, Kasai S, Yahathugoda TC, Weerasooriya MV, Kimura E (2011). Development of loop-mediated isothermal amplification method for detecting Wuchereria bancrofti DNA in human blood and vector mosquitoes. Parasitol Int.

[60] Senyonjo L, Oye J, Bakajika D, Biholong B, Tekle A, Boakye D, Schmidt E, Elhassan E (2016). Factors associated with Ivermectin non-compliance and its potential role in sustaining Onchocerca volvulus transmission in the west region of Cameroon. PLoS Negl Trop Dis.

