# Correction to: Differential susceptibility of Onchocerca volvulus microfilaria to ivermectin in two areas of contrasting history of mass drug administration in Cameroon: relevance of microscopy and molecular techniques for the monitoring of skin microfilarial repopulation within six months of direct observed treatment

**DOI:** 10.1186/s12879-021-05797-2

**Published:** 2021-01-18

**Authors:** Raphael Awah Abong, Glory N. Amambo, Patrick W. Chounna Ndongmo, Abdel Jelil Njouendou, Manuel Ritter, Amuam Andrew Beng, Mathias Eyong Esum, Kebede Deribe, Jerome Fru-Cho, Fanny F. Fombad, Theobald Mue Nji, Peter Ivo Enyong, Catherine B. Poole, Kenneth Pfarr, Achim Hoerauf, Clotilde K. S. Carlow, Samuel Wanji

**Affiliations:** 1 Parasites and Vector Research Unit (PAVRU), Department of Microbiology and Parasitology, University of Buea, P.O. Box 63, Buea, Cameroon; 2 Research Foundation in Tropical Diseases and Environment (REFOTDE), P.O. Box 474, Buea, Cameroon; 3 Department of Biomedical science, Faculty of Health Sciences, University of Buea, P.O. Box 63, Buea, Cameroon; 4 Institute for Medical Microbiology, Immunology and Parasitology, University Hospital Bonn, Bonn, Germany; 5 Global Health and Infection Department, Brighton and Sussex Medical School, Brighton, BN1 9PX UK; 6 School of Public Health, Addis Ababa University, Addis Ababa, Ethiopia; 7 Department of Sociology and Anthropology, University of Buea, Buea, Cameroon; 8 New England Biolabs, Ipswich, MA USA; 9 German Center for Infection Research (DZIF), partner site Bonn-Cologne, Bonn, Germany

**Correction to: BMC Infect Dis 20, 726 (2020)**

**https://doi.org/10.1186/s12879-020-05444-2**

After publication of the original article [1], the authors identified an error in the author name of Manuel Ritter. The given name and family name were erroneously transposed.

The incorrect author name is:

<GivenName>Ritter</GivenName>

<FamilyName>Manuel</FamilyName>

The correct author name is:

<GivenName>Manuel</GivenName>.

<FamilyName>Ritter</FamilyName>.

The original article has been corrected.
