# Supplementary material for: Differential susceptibility of Onchocerca volvulus microfilaria to ivermectin in two areas of contrasting history of mass drug administration in Cameroon: relevance of microscopy and molecular techniques for the monitoring of skin microfilarial repopulation within six months of direct observed treatment
Source: BMC Infect Dis. 2020 Oct 2;20:726. doi: 10.1186/s12879-020-05444-2 (PMC7530974; doi:10.1186/s12879-020-05444-2)
Supplement: Supplementary file 1 — Additional file 1 S1 Fig Check for inhibitory factors in DNA. [file 12879_2020_5444_MOESM1_ESM.pdf]

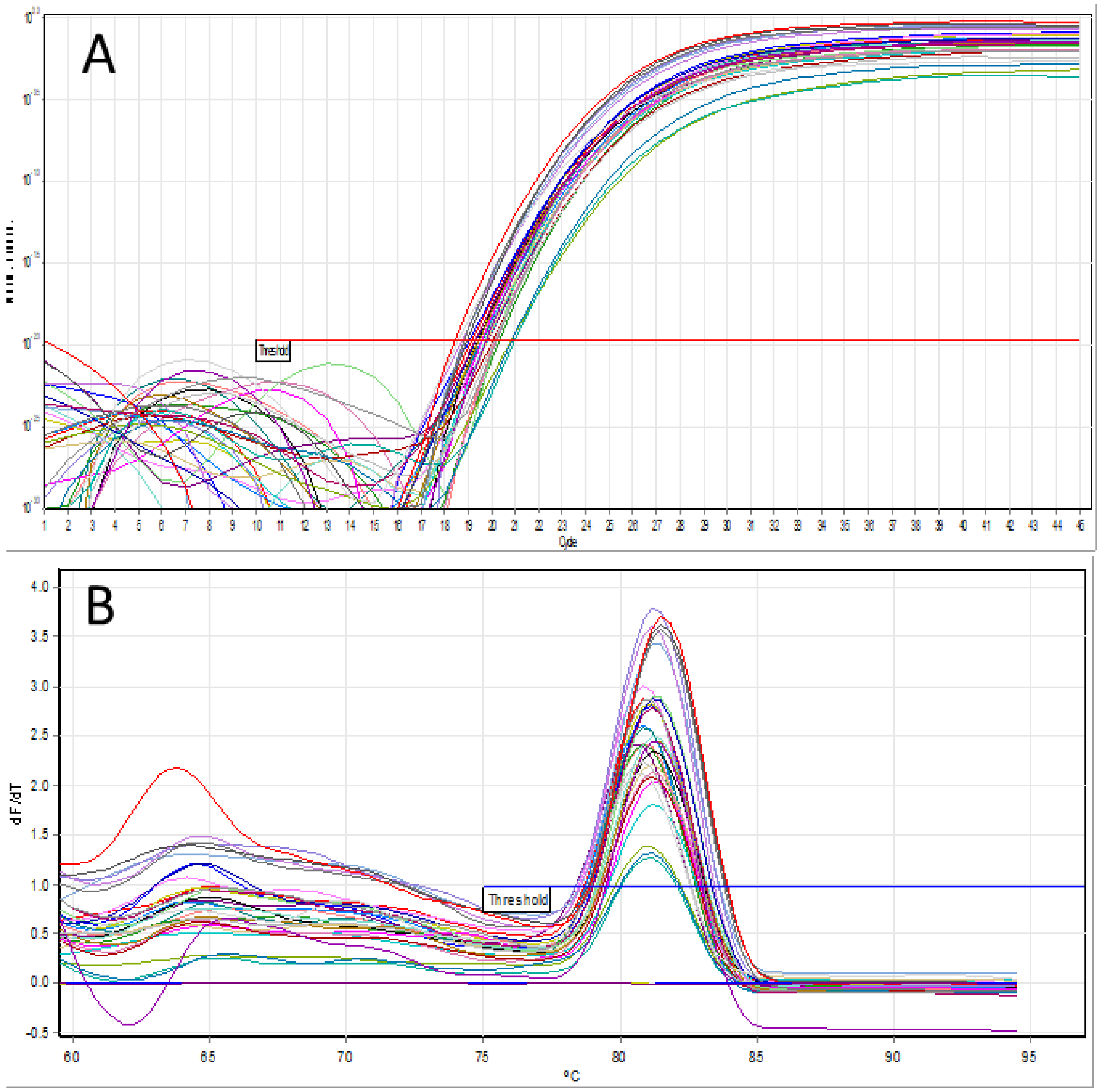

Figure S1. Representative analysis of PCR inhibitors in DNA samples. A real-time PCR that detects mouse interferon-γ plasmid spiked into the PCR master mix was used to determine if there were PCR inhibitors in the extracted DNA as published [1, 2]. Samples that deviated more than 3 cycles form the expected Ct of 18.76 (plasmid alone) would be considered to contain inhibitors and would be diluted stepwise 1:10 until the inhibition was no longer seen. B) Melting curve analysis of samples to confirm that the PCR product was the expected mouse interferon-γ sequence. For all samples tested, no evidence of inhibition was seen.

1. Batsa Debrah L, Phillips RO, Pfarr K, Klarmann-Schulz U, Opoku VS, Nausch N, et al. The efficacy of doxycycline treatment on *Mansonella perstans* infection: An open-label, randomized trial in Ghana. Am J Trop Med Hyg. 2019. doi: 10.4269/ajtmh.18-0491. PubMed PMID: 31162017.
2. Colebunders R, Mandro M, Mokili JL, Mucinya G, Mambandu G, Pfarr K, et al. Risk factors for epilepsy in Bas-Uele Province, Democratic Republic of the Congo: a case-control study. Int J Infect Dis. 2016;49:1-8. doi: 10.1016/j.ijid.2016.05.018. PubMed PMID: 27210267.
