# Supplementary material for: Differential susceptibility of Onchocerca volvulus microfilaria to ivermectin in two areas of contrasting history of mass drug administration in Cameroon: relevance of microscopy and molecular techniques for the monitoring of skin microfilarial repopulation within six months of direct observed treatment
Source: BMC Infect Dis. 2020 Oct 2;20:726. doi: 10.1186/s12879-020-05444-2 (PMC7530974; doi:10.1186/s12879-020-05444-2)
Supplement: Supplementary file 2 — Additional file 2 S2 Fig Detection limits of OvActin real-time PCR. [file 12879_2020_5444_MOESM2_ESM.pdf]

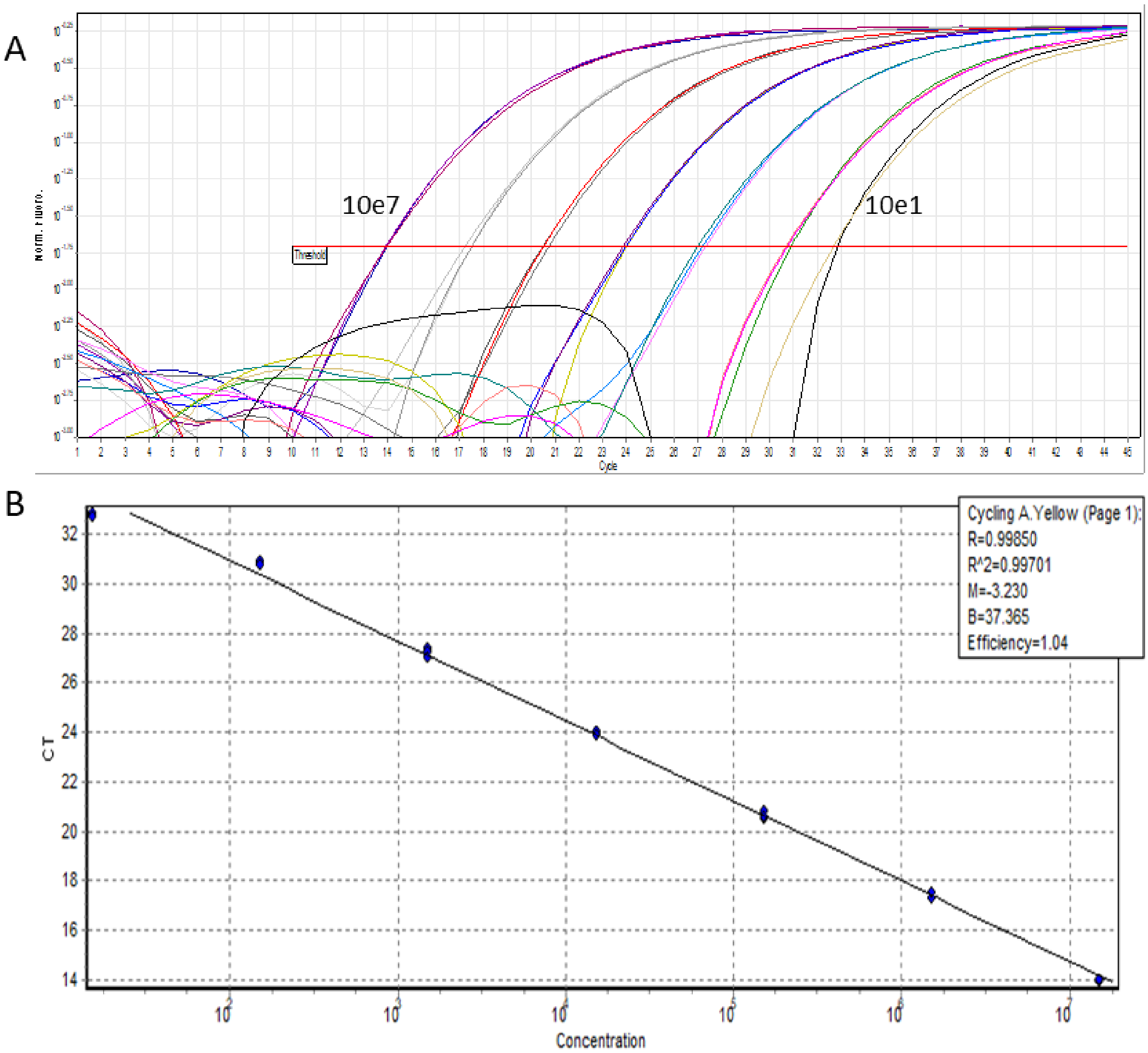

Figure S2 Fig. Detection limit of OvActin real-time PCR. A standard curve of plasmid containing the Ov actin PCR fragment was serially diluted 1:10 and run in a Rotor-Gene. A) The lowest dilution in which two of the three replicates had the same C_t was selected as the limit of detection for the real-time PCR. Thus, the OcActin real-time PCR reaction had a limit of detection of 10 copies/µL. B) Efficiency of the standard curve was >95%.
