# Supplementary figures and images for: Differential susceptibility of Onchocerca volvulus microfilaria to ivermectin in two areas of contrasting history of mass drug administration in Cameroon: relevance of microscopy and molecular techniques for the monitoring of skin microfilarial repopulation within six months of direct observed treatment
Source: BMC Infect Dis. 2020 Oct 2;20:726. doi: 10.1186/s12879-020-05444-2 (PMC7530974; doi:10.1186/s12879-020-05444-2)

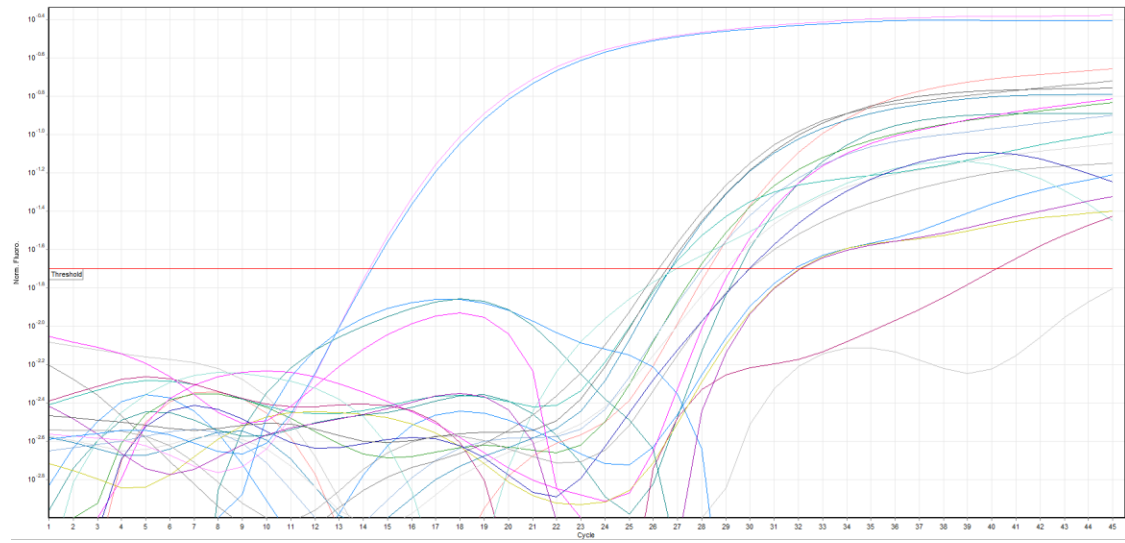

Supplement: Supplementary file 3 — Additional file 3 S3 Fig Representative data for a 45-cycle reaction for the duplex real-time PCR assay showing positive (above threshold level indicated by the red horizontal line) and negative (below threshold level) signals. [file 12879_2020_5444_MOESM3_ESM.pdf]

**A**

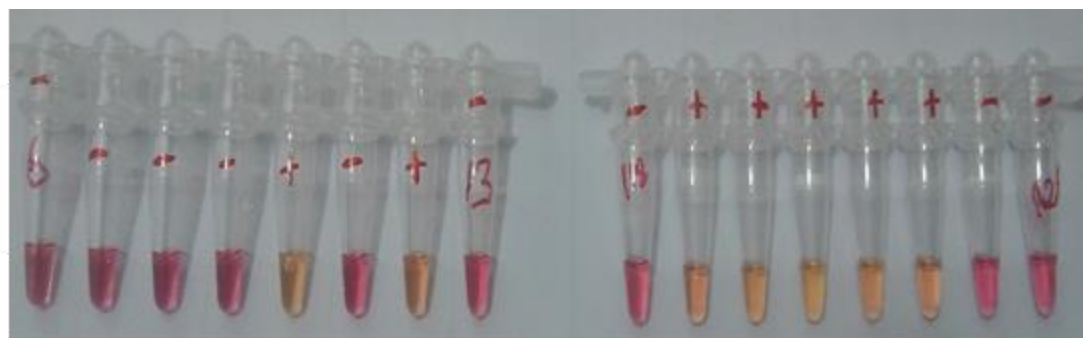

**B**

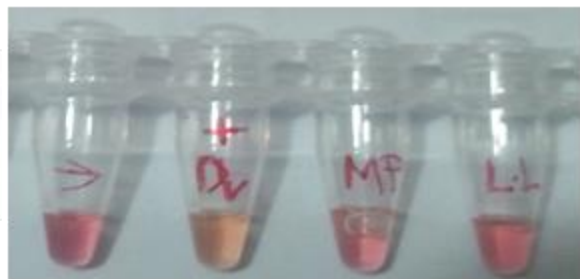

Supplement: Supplementary file 4 — Additional file 4 S4 Fig. A. Representative Data obtained from individual samples using a colorimetric LAMP assay. Samples containing O. volvulus microfilariae turned yellow (+) and were scored positive. Negative skin snips samples remained pink (−). B. Specificity of colorimetric LAMP assay. Reactions contained no template DNA (>) or DNA from Onchocerca volvulus (OV), Mansonella perstans (MP) or L. loa (LL). [file 12879_2020_5444_MOESM4_ESM.pdf]
