# Supplementary material for: Differential susceptibility of Onchocerca volvulus microfilaria to ivermectin in two areas of contrasting history of mass drug administration in Cameroon: relevance of microscopy and molecular techniques for the monitoring of skin microfilarial repopulation within six months of direct observed treatment
Source: BMC Infect Dis. 2020 Oct 2;20:726. doi: 10.1186/s12879-020-05444-2 (PMC7530974; doi:10.1186/s12879-020-05444-2)
Supplement: Supplementary file 5 — Additional file 5 S1 Table. Data input for the simplified interface of three-tests in one-population model (Walter and Irwig model) in Bafia and Melong Health Districts. [file 12879_2020_5444_MOESM5_ESM.doc]

**S1 Table:** Statistics of data input into the three-tests in one-population Web-Based interface for analysis.

| **Data Input** | | | | | | | | | | | | |
| --- | --- | --- | --- | --- | --- | --- | --- | --- | --- | --- | --- | --- |
|  | | | | **Bafia HD** | | | |  | **Melong HD** | | | |
|  |  |  |  | **D30** | **D90** | **D180** | **All** |  | **D30** | **D90** | **D180** | **All** |
| **SN** | **Test A** **(Microscopy)** | **Test B** **(real-time PCR)** | **Test C** **(LAMP)** | Observed frequency | | | |  | Observed frequency | | | |
| **1** | Positive | Positive | Positive | 10 | 11 | 21 | **42** |  | 2 | 3 | 8 | **13** |
| **2** | Positive | Positive | Negative | 0 | 0 | 0 | **0** |  | 0 | 0 | 0 | **0** |
| **3** | Positive | Negative | Positive | 1 | 0 | 0 | **1** |  | 0 | 4 | 0 | **4** |
| **4** | Negative | Positive | Positive | 1 | 4 | 5 | **10** |  | 2 | 0 | 1 | **3** |
| **5** | Positive | Negative | Negative | 0 | 1 | 1 | **2** |  | 0 | 2 | 1 | **3** |
| **6** | Negative | Positive | Negative | 1 | 1 | 0 | **2** |  | 0 | 0 | 0 | **0** |
| **7** | Negative | Negative | Positive | 3 | 4 | 5 | **12** |  | 3 | 4 | 7 | **14** |
| **8** | Negative | Negative | Negative | 35 | 30 | 16 | **81** |  | 22 | 31 | 21 | **74** |
