# Supplementary material for: Differential susceptibility of Onchocerca volvulus microfilaria to ivermectin in two areas of contrasting history of mass drug administration in Cameroon: relevance of microscopy and molecular techniques for the monitoring of skin microfilarial repopulation within six months of direct observed treatment
Source: BMC Infect Dis. 2020 Oct 2;20:726. doi: 10.1186/s12879-020-05444-2 (PMC7530974; doi:10.1186/s12879-020-05444-2)
Supplement: Supplementary file 6 — Additional file 6 S2 Table. Socio-demographic characteristics and distribution of participants screened by microscopy in the Bafia Health District. [file 12879_2020_5444_MOESM6_ESM.doc]

**S2 Table:**.Socio-demographic characteristics and distribution of participants screened by microscopy in the Bafia Health District

| **Demographic variables** | | **Number examined** | **Percentage (%)** |
| --- | --- | --- | --- |
| **Sex** | Male | 230 | 59.3 |
|  | Female | 158 | 40.7 |
|  | **Total** | **388** | **100** |
| **Age-group** | Children (5- 19 years) | 77 | 19.8 |
|  | Adults (≥20 years) | 311 | 80.1 |
|  | **Total** | **388** | **100** |
| **Communities** | Balamba 1 | 28 | 7.2 |
|  | Balamba 2 | 57 | 14.7 |
|  | Biamo | 68 | 17.5 |
|  | Botatango/Boalondo | 53 | 13.7 |
|  | Lable/Nyamsong | 88 | 22.7 |
|  | Ngomo/Biatsota | 94 | 24.2 |
|  | **Total** | **388** | **100** |
|  |  |  |  |
