# Supplementary material for: Differential susceptibility of Onchocerca volvulus microfilaria to ivermectin in two areas of contrasting history of mass drug administration in Cameroon: relevance of microscopy and molecular techniques for the monitoring of skin microfilarial repopulation within six months of direct observed treatment
Source: BMC Infect Dis. 2020 Oct 2;20:726. doi: 10.1186/s12879-020-05444-2 (PMC7530974; doi:10.1186/s12879-020-05444-2)
Supplement: Supplementary file 7 — Additional file 7 S3 Table. Socio-demographic characteristics and distribution of participants screened by microscopy in the Melong health district. [file 12879_2020_5444_MOESM7_ESM.doc]

**S3 Table.** Socio-demographic characteristics and distribution of participants screened by microscopy in the Melong health district.

| **Demographic variables** | | **Number examined** | **Percentage (%)** |
| --- | --- | --- | --- |
| **Sex** | Male | 283 | 47.2 |
|  | Female | 316 | 52.8 |
|  | **Total** | **599** | **100** |
| **Age-group** | Children (5- 19 years) | 132 | 22 |
|  | Adults (≥20 years) | 467 | 78 |
|  | **Total** | **599** | **100** |
| **Communities** | Mounko | 60 | 10.0 |
|  | Manjibo | 51 | 8.5 |
|  | Singa/Mbie/Barembeng2/Longze | 104 | 17.3 |
|  | Ndoumbot/Ntangtom | 55 | 9.2 |
|  | Nkoniakoniama/Nkonianke/Nkoniambot | 87 | 14.5 |
|  | Ndom-Bakem | 79 | 13.2 |
|  | Barembeng1 | 101 | 16.9 |
|  | Mpaka | 62 | 10.4 |
|  | **Total** | **599** | **100** |
