# Supplementary material for: Differential susceptibility of Onchocerca volvulus microfilaria to ivermectin in two areas of contrasting history of mass drug administration in Cameroon: relevance of microscopy and molecular techniques for the monitoring of skin microfilarial repopulation within six months of direct observed treatment
Source: BMC Infect Dis. 2020 Oct 2;20:726. doi: 10.1186/s12879-020-05444-2 (PMC7530974; doi:10.1186/s12879-020-05444-2)
Supplement: Supplementary file 8 — Additional file 8 S4 Table. Socio-demographic characteristics and distribution of participants that volunteered for the follow up study in the Bafia health district. [file 12879_2020_5444_MOESM8_ESM.doc]

**S4 Table:** Socio-demographic characteristics and distribution of participants that volunteered for the follow up study in the Bafia health district

| **Demographic variables** | | **Number examined** | **Percentage (%)** |
| --- | --- | --- | --- |
| **Sex** | Male | 40 | 78.4 |
|  | Female | 11 | 21.6 |
|  | **Total** | **51** | **100** |
| **Age-group** | Children (5- 19 years) | 1 | 2.0 |
|  | Adults (≥20 years) | 50 | 98.0 |
|  | **Total** | **51** | **100** |
|  | Balamba 1 | 4 | 7.8 |
|  | Balamba 2 | 2 | 3.9 |
|  | Biamo | 12 | 23.3 |
| **Communities** | Botatango/Boalondo | 9 | 17.6 |
|  | Lable/Nyamsong | 6 | 11.8 |
|  | Ngomo/Biatsota | 18 | 35.3 |
|  | **Total** | **51** | **100** |
