# Supplementary material for: Differential susceptibility of Onchocerca volvulus microfilaria to ivermectin in two areas of contrasting history of mass drug administration in Cameroon: relevance of microscopy and molecular techniques for the monitoring of skin microfilarial repopulation within six months of direct observed treatment
Source: BMC Infect Dis. 2020 Oct 2;20:726. doi: 10.1186/s12879-020-05444-2 (PMC7530974; doi:10.1186/s12879-020-05444-2)
Supplement: Supplementary file 9 — Additional file 9 S5 Table. Socio-demographic characteristics and distribution of participants that volunteered for the follow up study in the Melong health district. [file 12879_2020_5444_MOESM9_ESM.doc]

**S5 Table:** Socio-demographic characteristics and distribution of participants that volunteered for the follow up study in the Melong health district

| **Demographic variables** | | **Number examined** | **Percentage (%)** |
| --- | --- | --- | --- |
| **Sex** | Male | 26 | 59.1 |
|  | Female | 18 | 40.1 |
|  | **Total** | **44** | **100** |
| **Age-group** | Children (5- 19 years) | 6 | 13.6 |
|  | Adults (≥20 years) | 38 | 86.4 |
|  | **Total** | **44** | **100** |
| **Communities** | Mounko | 4 | 9.1 |
|  | Manjibo | 8 | 18.2 |
|  | Singa/Mbie/Barembeng2/Longze | 7 | 15.9 |
|  | Ndoumbot/Ntangtom | 4 | 9.1 |
|  | Nkoniakoniama/Nkonianke/Nkoniambot | 5 | 11.4 |
|  | Ndom-Bakem | 6 | 13.6 |
|  | Barembeng1 | 6 | 13.6 |
|  | Mpaka | 4 | 9.1 |
|  | **Total** | **44** | **100** |
